# Gut Microbiota in Children With Cystic Fibrosis: A Taxonomic and Functional Dysbiosis

**DOI:** 10.1038/s41598-019-55028-7

**Published:** 2019-12-09

**Authors:** Michael J. Coffey, Shaun Nielsen, Bernd Wemheuer, Nadeem O. Kaakoush, Millie Garg, Bronwen Needham, Russell Pickford, Adam Jaffe, Torsten Thomas, Chee Y. Ooi

**Affiliations:** 1 Discipline of Paediatrics, School of Women’s and Children’s Health, University of New South Wales, Sydney, NSW Australia; 2 Centre for Marine Science and Innovation, School of Biological, Earth and Environmental Sciences, University of New South Wales, Sydney, NSW Australia; 3 School of Medical Sciences, University of New South Wales, Sydney, NSW Australia; 4 Bioanalytical Mass Spectrometry Facility, Mark Wainwright Analytical Centre (MWAC), University of New South Wales, Sydney, NSW Australia; 5 Molecular and Integrative Cystic Fibrosis (miCF) Research Centre, High Street, Randwick, NSW Australia; 6 Department of Respiratory, Sydney Children’s Hospital, High Street, Randwick, NSW Australia; 7 Department of Gastroenterology, Sydney Children’s Hospital, High Street, Randwick, NSW Australia

**Keywords:** Cystic fibrosis, Dysbiosis

## Abstract

Intestinal dysbiosis has been observed in children with cystic fibrosis (CF), yet the functional consequences are poorly understood. We investigated the functional capacity of intestinal microbiota and inflammation in children with CF. Stool samples were collected from 27 children with CF and 27 age and gender matched healthy controls (HC) (aged 0.8–18 years). Microbial communities were investigated by iTag sequencing of 16S rRNA genes and functional profiles predicted using Tax4Fun. Inflammation was measured by faecal calprotectin and M2-pyruvate kinase. Paediatric CF gastrointestinal microbiota demonstrated lower richness and diversity compared to HC. CF samples exhibited a marked taxonomic and inferred functional dysbiosis when compared to HC. In children with CF, we predicted an enrichment of genes involved in short-chain fatty acid (SCFA), antioxidant and nutrient metabolism (relevant for growth and nutrition) in CF. The notion of pro-inflammatory GI microbiota in children with CF is supported by positive correlations between intestinal inflammatory markers and both genera and functional pathways. We also observed an association between intestinal genera and both growth z-scores and FEV1%. These taxonomic and functional changes provide insights into gastrointestinal disease in children with CF and future gastrointestinal therapeutics for CF should explore the aforementioned pathways and microbial changes.

## Introduction

Gastrointestinal (GI) disease in cystic fibrosis (CF) begins *in utero*, continues throughout childhood and into adulthood^1,2^. Dysfunction of the cystic fibrosis transmembrane conductance regulator (CFTR) results in an altered intestinal milieu with proposed disease factors including: (i) reduced bicarbonate secretion and low intestinal pH, (ii) thick and inspissated mucus, (iii) a lack of endogenous pancreatic enzymes, (iv) delayed intestinal transit and (v) possibly an impaired innate immunity^3,4^. These mechanisms in combination with an energy- and fat-dense diet^5^ and frequent antibiotic usage likely contribute to a dysbiosis of the intestinal bacterial community, which has been observed in CF^6–9^. Individuals with CF suffer from maldigestion and malabsorption, which contribute to malnutrition. Nutrition is of paramount importance in CF, as it has prognostic value for growth, lung function, overall well-being and mortality^10^.

Intestinal inflammation has been reported in several studies indirectly using various biomarkers, including calprotectin, rectal nitric oxide and M2-pyruvate kinase (M2-PK), as well as directly by capsule endoscopy^11–16^. The pathogenesis of intestinal inflammation in CF is poorly understood, however the altered intestinal environment and microbiota likely play a role. Intestinal inflammation in CF may have significant clinical relevance due to its link with growth and nutrition^11,17,18^, and to the increased risk of GI cancers in the adult patient population^19–21^.

Recently, Manor *et al*.^8^ utilised metagenomic shotgun sequencing of faecal samples and suggested that in children with CF, enteric fat abundance selects for a pro-inflammatory GI microbiota. Debyser *et al*.^7^ utilised liquid chromatography-mass spectrometry (LC-MS) on faecal protein extracts from children with CF and their unaffected siblings to highlight the presence of an intestinal dysbiosis and inflammation in CF. Vernocchi *et al*.^9^ utilised 16S rRNA sequencing and metabolomics (LC-MS) in children with CF and age-matched healthy controls (HC) to suggest that the gut microbiota enterophenotypes of CF, together with endogenous and bacterial CF biomarkers, reflect the expression of functional alteration at the intestinal level.

These studies all demonstrate an altered functional environment and with established and recent tools for microbial modulation (e.g. prebiotics, probiotics, synbiotics, antibiotics, diet and faecal microbiota transplantation), understanding these changes in the CF gut is essential for developing potential therapeutics. It is therefore essential to explore and define the functional differences between children with CF and well-matched HC. Furthermore, the links between intestinal bacteria and markers of inflammation have yet to be characterised in CF.

In this study we investigate composition and function of bacterial communities inhabiting the intestines of children with CF. We hypothesise that the composition and functional capacity of bacterial communities in the intestine of children with CF are different when compared to HC. We also hypothesise that intestinal bacteria and their functional profiles will be associated with biomarkers of intestinal inflammation. Furthermore, we hypothesise that these changes will be associated with growth and lung function in children with CF.

## Results

### Population characteristics

Initially, 39 children with CF and 39 paired HC (matched for age and gender) were included, whose microbiota was assessed using 16S rRNA gene sequencing. After removal of low-coverage samples (sequencing depth <22,578, see below), the final study population comprised of 27 children with CF and 27 paired. The mean (SD) age of CF and HC subjects was 8.2 (5.0) and 7.7 (5.3) years respectively (p = 0.2). Each group consisted of 14 males (51.9%). Of the CF children, 24 were pancreatic insufficient (PI) and 3 pancreatic sufficient (PS). Twenty-two of the CF children were homozygous F508del (81%), whilst the remaining 5 were heterozygotes. Clinical characteristics are presented in Table 1.

**Table 1:** Clinical characteristics of included participants.

ID	Gender	Group	Pancreas	Age (yr)	Genotype	Calprotectin (mg/kg)	M2-PK (U/ml)
CF1	M	CF	PS	1.2	F508del/D1152H	101.3925	1
HC1	M	HC	—	0.8	−/−	<19.5325	—
CF3	F	CF	PI	1.5	F508del/F508del	27.915	8.029
HC3	F	HC	—	0.9	−/−	<19.5325	1
CF4	F	CF	PI	1.6	F508del/F508del	66.19	13.984
HC4	F	HC	—	1.3	−/−	<19.5325	1
CF5	M	CF	PI	2.7	F508del/F508del	60.545	3.814
HC5	M	HC	—	1.9	−/−	<19.5325	1
CF7	M	CF	PI	3.1	F508del/F508del	56.7975	35.035
HC7	M	HC	—	3.8	−/−	<19.5325	1
CF11	F	CF	PI	3.6	F508del/F508del	124.7	101.03
HC11	F	HC	—	2.3	−/−	20.0775	1
CF12	M	CF	PI	3.8	F508del/F508del	203.7725	203.95
HC12	M	HC	—	3.9	−/−	32.5975	1
CF13	M	CF	PI	3.9	F508del/F508del	<19.5325	7.147
HC13	M	HC	—	4.5	−/−	—	—
CF14	M	CF	PI	4.4	F508del/F508del	157.485	48.4625
HC14	M	HC	—	4.7	−/−	<19.5325	1
CF15	F	CF	PI	4.9	F508del/W1282X	61.3075	5.875
HC15	F	HC	—	2.4	−/−	24.9825	1
CF17	M	CF	PI	5.0	F508del/F508del	40.185	38.6475
HC17	M	HC	—	5.1	−/−	<19.5325	1
CF20	F	CF	PI	5.8	F508del/F508del	171.4025	6.133
HC20	F	HC	—	3.7	−/−	24.23	—
CF22	F	CF	PI	7.0	F508del/F508del	55.865	9.128
HC22	F	HC	—	5.0	−/−	—	—
CF25	M	CF	PI	8.2	F508del/F508del	<19.5325	50
HC25	M	HC	—	8.6	−/−	—	—
CF26	F	CF	PI	9.8	F508del/F508del	<19.5325	13.553
HC26	F	HC	—	6.9	−/−	—	—
CF27	F	CF	PI	9.8	F508del/F508del	106.29	3.962
HC27	F	HC	—	7.6	−/−	—	—
CF28	M	CF	PI	10.8	F508del/R334W	48.1175	1.397
HC28	M	HC	—	11.6	−/−	—	—
CF29	F	CF	PI	11.0	F508del/F508del	47.4175	3.227
HC29	F	HC	—	8.2	−/−	<19.5325	1
CF31	M	CF	PI	11.6	F508del/F508del	92	12.938
HC31	M	HC	—	11.7	−/−	<19.5325	1
CF32	M	CF	PI	11.7	F508del/F508del	50.775	52.43
HC32	M	HC	—	13.9	−/−	—	—
CF33	F	CF	PS	12.3	F508del/unknown	33.0875	1
HC33	F	HC	—	10.5	−/−	<19.5325	1
CF34	F	CF	PI	12.4	F508del/F508del	49.145	14.923
HC34	F	HC	—	11.7	−/−	—	—
CF35	M	CF	PI	13.0	F508del/F508del	360.3575	3.181
HC35	M	HC	—	15.1	−/−	<19.5325	1
CF36	M	CF	PS	14.0	F508del/V232D	127.1925	5.493
HC36	M	HC	—	18.1	−/−	<19.5325	3.264
CF37	F	CF	PI	16.0	F508del/F508del	645.5825	4.458
HC37	F	HC	—	11.8	−/−	<19.5325	1.816
CF38	M	CF	PI	16.1	F508del/F508del	95.7875	50
HC38	M	HC	—	18.2	−/−	27.675	1
CF39	F	CF	PI	17.3	F508del/F508del	80.0575	138.93
HC39	F	HC	—	14.3	−/−	<19.5325	1

### Bacterial community characteristics

A total of 3,179,543 bacterial 16S rRNA sequences were retrieved covering more than 72% of the bacterial diversity per sample. This is further supported by the rarefaction curves, which shows that the majority of bacterial communities in our samples are being covered by the sequencing effort undertaken here (Supp. Fig. 1).

### Alpha diversity

**Figure 1:**
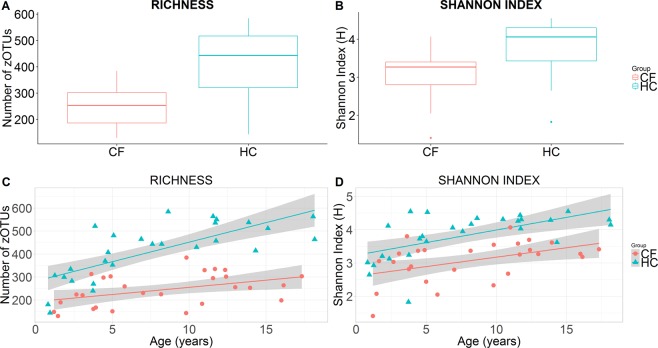
Boxplots of sample richness (number of zOTUs) (**A**) and Shannon index (**B**) in CF and HC cohorts. Scatterplots of sample richness (number of zOTUs) (**C**) and Shannon index (**D**) against age in CF and HC cohorts. Cohort mean and 95% confidence intervals are constructed from generalised linear models and presented as lines and shaded regions, respectively (**C**,**D**).

Children with CF compared with HC had a significantly lower bacterial richness as assessed by the number of zOTUs (mean difference (95% CI) of −168.8 (−219.5 to −118.1), p = 2.9 × 10^−07^) and a lower diversity as assessed by the Shannon index (mean difference (95% CI) of −0.74 (−1.00 to −0.49), p = 2.2 × 10^−06^) (Fig. 1A,B). Controlling for age, children with CF had consistently lower richnesses (estimate (SE) −175.1 (21.6), p = 1.0 × 10^−10^) and Shannon diversity indices (estimate (SE) −0.78 (0.15), p = 3.2 × 10^−06^) (Fig. 1C,D).

### Phylogeny-based beta diversity

**Figure 2:**
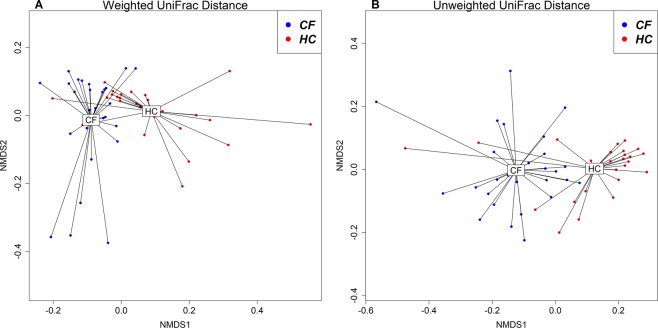
NMDS plots based on weighted (**A**) and unweighted (**B**) UniFrac distances between CF and HC cohorts.

Visualisation of phylogeny-based beta diversity (weighted and unweighted UniFrac distances) is presented in Fig. 2. PERMANOVA showed a significant difference in bacterial communities between the CF and HC cohorts (weighted UniFrac distance R^2^ = 0.139, p = 0.001, unweighted UniFrac distance R^2^ = 0.121, p = 0.001). PERMANOVA showed no significant difference in bacterial communities between males and females (weighted UniFrac distance R^2^ = 0.029, p = 0.2, unweighted UniFrac distance R^2^ = 0.018, p = 0.5), or with age (weighted UniFrac distance R^2^ = 0.013, p = 0.7, unweighted UniFrac distance R^2^ = 0.026, p = 0.09).

### Differences in microbial communities between CF and HC populations

**Figure 3:**
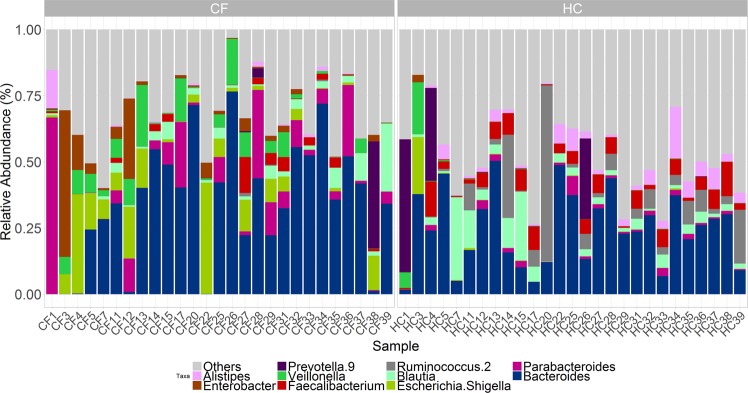
Relative abundance of top 10 most abundant bacterial genera for CF and HC subjects. Samples ordered in increasing age (from left to right).

The relative abundance of all bacterial phyla in CF and HC subjects are presented in Supplementary Fig. 2. Relative abundances of the top 10 most abundant bacterial genera in CF and HC are presented in Fig. 3. Bacterial taxa with a significant different abundance (ANCOM with q < 0.05) between CF and HC populations are presented in Table 2 and Supplementary Fig. 3.

**Table 2:** Bacterial taxa with a significant different abundance (ANCOM with q < 0.05) between CF and HC populations at each taxonomic rank (bold). Arrows indicate if the relative abundance of each taxa is higher (**↑**) or lower (**↓**) in CF compared with HC populations. Taxa without an arrow describe phylogeny and were not significantly different in abundance. ANCOM analysis presented in Supplementary Fig. 3.

Phylum	Class	Order	Family	Genus
↑ Fusobacteria	Fusobacteriia	Fusobacteriales	Fusobacteriaceae	↑ *Fusobacterium*
↑ Proteobacteria	↑ Gammaproteobacteria	↑ Enterobacteriales	↑ Enterobacteriaceae	↑ *Enterobacter*
				↑ *Escherichia*, *Shigella*
				↑ *Morganella*
↓ Verrucomicrboia	↓ Verrucomicrobiae	↓ Verrucomicrobiales	↓ Verrucomicrobiaceae	
Firmicutes	↑ Negativicutes	↑ Selenomonadales	↑ Veillonellaceae	↑ *Veillonella*
	Mollicutes	↓ Mollicutes RF9	↓ uncultured Mollicutes RF9	
	Clostridia	Clostridiales	↓ Christensenellaceae	↓ *Christensenellaceae R7* group
			↓ Family XIII	
			↓ Ruminococcaceae	↓ *Ruminiclostridium* 6
				↓ *Ruminococcaceae UCG* 002
				↓ *Ruminococcaceae UCG* 005
				↓ *Ruminococcaceae UCG* 013
				↓ *Ruminococcaceae UCG* 014
				↓ *Ruminococcus* 1
				↓ *Ruminococcus* 2
				↓ *Eubacterium coprostanoligenes* group
			Eubacteriaceae	↓ *Eubacterium eligens* group
			Lachnospiraceae	↓ *Lachnospira*
				↓ *Lachnospiraceae NK*4*A136* group
				↑ *Ruminococcus gnavus* group
				↓ *Eubacterium ventriosum* group
				↓ *Eubacterium xylanophilum* group
				↑ *Tyzzerella 4*
	Bacilli	Lactobacillales	↑ Enterococcaceae	↑ *Enterococcus*
	Erysipelotrichia	Erysipelotrichales	Erysipelotrichaceae	↓ *Erysipelotrichaceae UCG* 003
				↑ *Clostridium innocuum* group
Bacteroidetes	Bacteroidia	Bacteroidales	↓ Rikenellaceae	↓ *Alistipes*

### Predicted functional analysis using Tax4Fun

**Figure 4:**
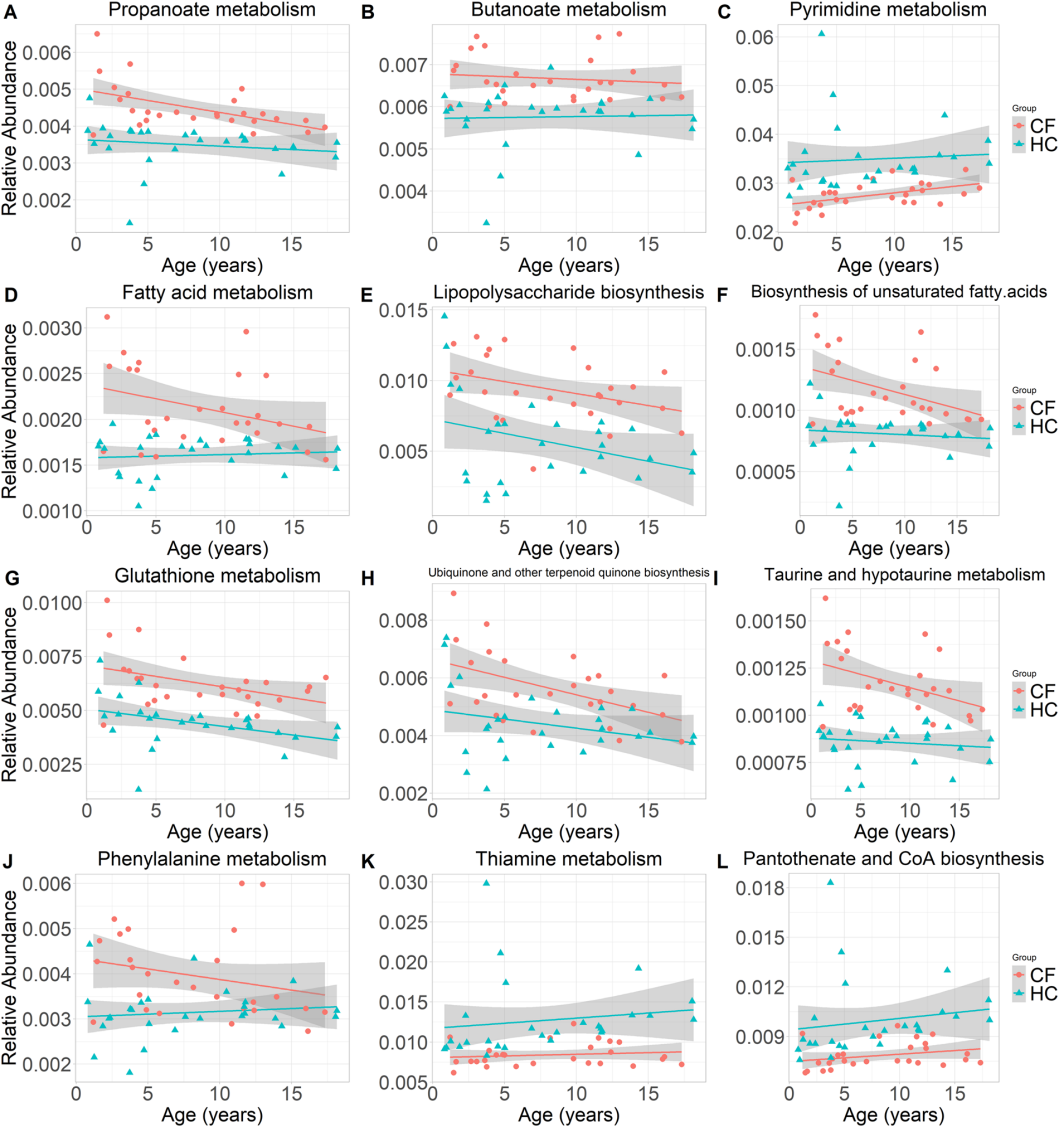
Scatterplots of the relative abundance of predicted KEGG pathways against age in CF and HC cohorts. Predicted functions associated with: (i) short-chain fatty acids (**A**,**B**), (ii) fatty acid metabolism (**C**–**F**), (iii) antioxidants (**G**–**I**), (iv) essential amino acid (**J**), and (v) vitamins (**K**–**L**). Cohort mean and 95% confidence intervals are constructed from generalised linear models and presented as lines and shaded regions, respectively.

To predict the functional profiles of intestinal bacteria, we used Tax4Fun. On average per sample, 84.6% of zOTUs could be used by Tax4Fun to predict a functional profile of the community based on KEGG Orthology (KO) terms, demonstrating high predictive power in our analysis. In total, abundances of 275 unique KO pathways (based on 6,286 unique functions) were predicted in CF and HC populations. The abundance of 56 KO pathways was significantly different between CF and HC pairs (p < 0.00019) (Table 3). Exploring the effect of age, linear models of pathways of interest were constructed and presented in Fig. 4.

**Table 3:** Predicted KEGG pathways with a significant different abundance between CF and HC pairs (p < 0.00019).

PATHWAY	KO	CF*	Association	CF Rel Abund Median % (IQR)	HC Rel Abund Median % (IQR)	Mean Difference** Est % (95% CI)	P-value
Pyrimidine metabolism	240	↓	Glucose, Fatty acids	2.78 (2.6–2.905)	3.3 (3.09–3.566)	−0.733 (−1.025–0.44)	8.32E-06
Alanine aspartate and glutamate metabolism	250	↓	Amino acids	1.41 (1.32–1.5)	1.78 (1.61–1.945)	−0.459 (−0.687–0.232)	1.88E-05
Thiamine metabolism	730	↓	Vitamins	0.792 (0.736–0.914)	1.14 (1.002–1.33)	−0.43 (−0.632–0.228)	1.31E-06
Lipopolysaccharide biosynthesis	540	↑	Fatty acids	0.913 (0.819–1.075)	0.54 (0.347–0.691)	0.365 (0.227–0.503)	3.52E-05
Pertussis	5133	↑	Bacteria	0.376 (0.226–0.669)	0.134 (0.093–0.164)	0.353 (0.217–0.489)	1.64E-06
Bacterial chemotaxis	2030	↓	Bacteria	0.615 (0.455–0.756)	0.922 (0.758–1.05)	−0.301 (−0.417–0.185)	1.35E-05
Cell cycle Caulobacter	4112	↓	Bacteria	1.31 (1.245–1.365)	1.56 (1.49–1.635)	−0.271 (−0.338–0.204)	5.93E-06
Pantothenate and CoA biosynthesis	770	↓	Vitamins	0.753 (0.74–0.831)	0.94 (0.857–1.015)	−0.209 (−0.307–0.111)	3.07E-05
Homologous recombination	3440	↓	DNA repair	1.61 (1.535–1.635)	1.72 (1.67–1.885)	−0.208 (−0.293–0.123)	3.97E-05
Glutathione metabolism	480	↑	Antioxidants	0.608 (0.556–0.651)	0.444 (0.401–0.477)	0.182 (0.13–0.233)	0.00002
Glyoxylate and dicarboxylate metabolism	630	↑	Glucose, Fatty acids	0.767 (0.723–0.912)	0.677 (0.617–0.696)	0.153 (0.101–0.205)	5.53E-06
Pyruvate metabolism	620	↑	Glucose, Fatty acids	1.19 (1.14–1.255)	1.08 (1.045–1.105)	0.143 (0.102–0.184)	9.29E-06
Ubiquinone and other terpenoid quinone biosynthesis	130	↑	Antioxidants	0.541 (0.488–0.631)	0.423 (0.379–0.486)	0.123 (0.074–0.171)	3.52E-05
Phenylalanine tyrosine and tryptophan biosynthesis	400	↓	Amino acids	1.15 (1.125–1.21)	1.24 (1.22–1.29)	−0.11 (−0.159–0.061)	9.64E-05
Citrate cycle TCA cycle	20	↑	Fatty acids, Glucose	0.553 (0.517–0.595)	0.444 (0.386–0.482)	0.109 (0.072–0.146)	6.66E-06
Vibrio cholerae pathogenic cycle	5111	↑	Bacteria	0.189 (0.163–0.33)	0.149 (0.112–0.164)	0.105 (0.061–0.149)	0.00014
Lysine degradation	310	↑	Amino acids	0.357 (0.331–0.387)	0.27 (0.237–0.293)	0.1 (0.073–0.128)	1.04E-07
Propanoate metabolism	640	↑	Fatty acids, SCFA	0.433 (0.415–0.471)	0.362 (0.339–0.383)	0.099 (0.073–0.125)	6.64E-06
Nicotinate and nicotinamide metabolism	760	↓	Vitamins	0.743 (0.718–0.774)	0.824 (0.797–0.903)	−0.093 (−0.135–0.05)	0.00013
Butanoate metabolism	650	↑	Fatty acids, SCFA	0.657 (0.624–0.692)	0.591 (0.57–0.608)	0.092 (0.058–0.125)	1.98E-05
Photosynthesis	195	↓	Bacteria	0.284 (0.26–0.304)	0.356 (0.318–0.375)	−0.084 (−0.116–0.052)	3.59E-05
Phenylalanine metabolism	360	↑	Amino acids	0.37 (0.322–0.452)	0.307 (0.295–0.337)	0.082 (0.044–0.119)	0.00018
Sulfur metabolism	920	↑	Amino acids	0.432 (0.399–0.45)	0.344 (0.305–0.379)	0.081 (0.055–0.108)	2.76E-05
Polycyclic aromatic hydrocarbon degradation	624	↓	Benzenes	0.23 (0.197–0.254)	0.292 (0.27–0.318)	−0.081 (−0.118–0.045)	1.35E-05
Valine leucine and isoleucine degradation	280	↑	Amino acids	0.383 (0.346–0.414)	0.313 (0.268–0.351)	0.077 (0.041–0.113)	0.00014
Protein processing in endoplasmic reticulum	4141	↓	Proteins	0.081 (0.066–0.099)	0.142 (0.116–0.166)	−0.075 (−0.111–0.04)	1.14E-05
D-Alanine metabolism	473	↓	Glucose	0.278 (0.256–0.316)	0.361 (0.33–0.379)	−0.071 (−0.096–0.045)	8.14E-05
Biosynthesis of siderophore group nonribosomal peptides	1053	↑	Iron	0.098 (0.082–0.138)	0.064 (0.055–0.07)	0.07 (0.041–0.099)	1.04E-06
Nitrotoluene degradation	633	↑	Benzenes	0.138 (0.104–0.184)	0.095 (0.085–0.115)	0.058 (0.029–0.087)	0.00013
Fatty acid metabolism	71	↑	Fatty acids	0.197 (0.183–0.252)	0.169 (0.144–0.174)	0.052 (0.034–0.07)	1.64E-06
Phosphonate and phosphinate metabolism	440	↑	Bacteria	0.168 (0.144–0.184)	0.113 (0.093–0.134)	0.048 (0.031–0.066)	8.14E-05
Peroxisome	4146	↑	Lipids	0.19 (0.176–0.207)	0.148 (0.127–0.164)	0.047 (0.028–0.065)	9.45E-05
Plant pathogen interaction	4626	↑	Bacteria	0.266 (0.253–0.278)	0.23 (0.215–0.243)	0.045 (0.024–0.067)	3.52E-05
Biosynthesis of unsaturated fatty acids	1040	↑	Fatty acids	0.102 (0.098–0.137)	0.085 (0.074–0.088)	0.036 (0.026–0.046)	1.49E-08
Tryptophan metabolism	380	↑	Amino acids	0.06 (0.054–0.117)	0.051 (0.044–0.054)	0.036 (0.022–0.05)	0.00002
Inositol phosphate metabolism	562	↑	Cellular function	0.204 (0.19–0.211)	0.174 (0.156–0.182)	0.034 (0.022–0.046)	3.78E-05
Carbon fixation in photosynthetic organisms	710	↓	Bacteria	0.429 (0.421–0.441)	0.456 (0.448–0.471)	−0.033 (−0.049–0.016)	0.00012
Taurine and hypotaurine metabolism	430	↑	Antioxidants	0.114 (0.104–0.132)	0.088 (0.082–0.092)	0.031 (0.024–0.038)	1.49E-08
Shigellosis	5131	↑	Bacteria	0.009 (0.003–0.024)	0.001 (0.0004–0.002)	0.026 (0.007–0.046)	2.98E-08
Toluene degradation	623	↑	Benzenes	0.075 (0.068–0.098)	0.059 (0.052–0.062)	0.023 (0.016–0.031)	6.41E-07
Bisphenol degradation	363	↑	Benzoates	0.196 (0.188–0.208)	0.176 (0.167–0.185)	0.023 (0.015–0.031)	5.15E-05
Glycosphingolipid biosynthesis lacto and neolacto series	601	↓	Cell membrane	0.018 (0.014–0.025)	0.036 (0.027–0.043)	−0.022 (−0.033–0.011)	4.75E-05
Chlorocyclohexane and chlorobenzene degradation	361	↑	Benzoates	0.051 (0.044–0.07)	0.041 (0.037–0.044)	0.018 (0.01–0.025)	1.88E-05
Penicillin and cephalosporin biosynthesis	311	↑	Antibiotics	0.076 (0.067–0.082)	0.06 (0.055–0.068)	0.017 (0.011–0.024)	4.57E-06
Phosphatidylinositol signaling system	4070	↑	Lipids	0.066 (0.058–0.07)	0.053 (0.046–0.058)	0.015 (0.008–0.021)	4.57E-06
Ethylbenzene degradation	642	↓	Benzenes	0.094 (0.086–0.098)	0.106 (0.102–0.111)	−0.014 (−0.02–0.008)	2.21E-05
Basal transcription factors	3022	↓	Transcription	0.013 (0.007–0.019)	0.024 (0.017–0.03)	−0.011 (−0.015–0.006)	1.59E-05
Fluorobenzoate degradation	364	↑	Benzoates	0.012 (0.01–0.024)	0.008 (0.005–0.009)	0.01 (0.006–0.014)	3.77E-06
Huntingtons disease	5016	↑	Genetic	0.031 (0.03–0.034)	0.024 (0.019–0.026)	0.009 (0.006–0.013)	3.41E-05
Type I diabetes mellitus	4940	↓	Autoimmune	0.04 (0.038–0.042)	0.047 (0.043–0.049)	−0.008 (−0.012–0.005)	1.25E-05
Amyotrophic lateral sclerosis ALS	5014	↑	Autoimmune	0.012 (0.009–0.017)	0.005 (0.004–0.007)	0.008 (0.006–0.011)	1.49E-08
alpha Linolenic acid metabolism	592	↑	Fatty acids	0.006 (0.004–0.009)	0.003 (0.003–0.004)	0.005 (0.003–0.007)	4.47E-08
Chagas disease American trypanosomiasis	5142	↑	Parasites	0.013 (0.011–0.014)	0.009 (0.006–0.011)	0.005 (0.002–0.008)	8.37E-05
VEGF signaling pathway	4370	↓	Blood vessel formation	0.0005 (0.0002–0.002)	0.005 (0.003–0.006)	−0.004 (−0.007–0.002)	1.59E-05
Calcium signaling pathway	4020	↓	Cell signalling	0.001 (0.001–0.003)	0.005 (0.003–0.007)	−0.004 (−0.007–0.002)	0.00013
Influenza A	5164	↓	Viruses	0.002 (0.002–0.003)	0.005 (0.004–0.006)	−0.003 (−0.005–0.002)	6.41E-07

Samples are ordered by absolute mean difference (decreasing). *Arrows indicate if the relative abundance of each pathway is higher (**↑**) or lower (↓) in CF compared with HC. **Estimate of mean difference between pairs based on Wilcoxon signed-rank testing. Est, estimate. KO, KEGG Orthology number. Rel Abund, relative abundance.

### Intestinal inflammation and its correlation with the bacterial community

**Figure 5:**
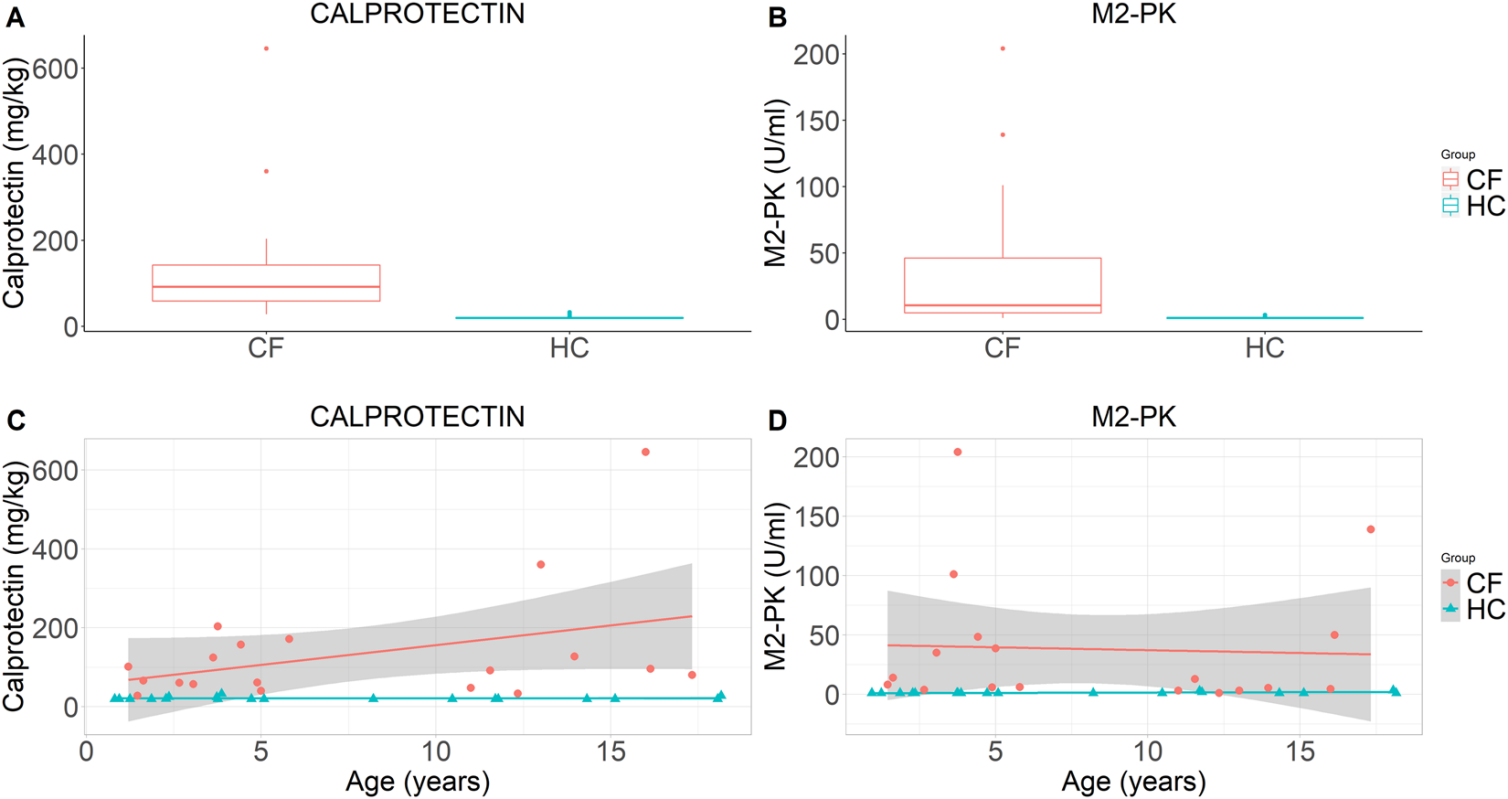
Boxplots of calprotectin (n = 19 pairs) and M2-PK (n = 18 pairs) in CF and HC cohorts (**A**,**B**). Scatterplots of calprotectin and M2-PK against age in CF and HC cohorts (**C**,**D**). Cohort mean and 95% confidence intervals are constructed from generalised linear models and presented as lines and shaded regions, respectively (**C**,**D**).

Faecal calprotectin levels were measured in 19/27 pairs (70.4%). Calprotectin levels were significantly elevated in CF compared with paired HC subjects (median (IQR) 92.0 mg/kg (58.7–142.3) vs. 19.5 mg/kg (19.5–19.8), respectively, p = 3.8 × 10^–6^) (Fig. 5A). Controlling for age, children with CF had consistently higher faecal calprotectin levels than HC (estimate (SE) 110.6 mg/kg (32.9), p = 0.0019) (Fig. 5C).

Faecal M2-PK levels were measured in 18/27 pairs (66.7%). M2-PK levels were significantly elevated in CF compared with paired HC subjects (median (IQR) 10.5 U/mL (4.7–46.0) vs. 1.0 U/mL (1.0–1.0) respectively, p = 0.00032) (Fig. 5B). Controlling for age, children with CF had consistently higher faecal M2-PK levels than HC (estimate (SE) 36.8 U/mL (13.4), p = 0.0096) (Fig. 5D).

Correlations between bacterial taxa (at each taxonomic rank) and inflammatory markers (calprotectin (n = 27) and M2-PK (n = 26)) within CF subjects are presented in Supplementary Fig. 4. Ten genera (*Acidaminococcus* (r = 0.8, q = 3.9 × 10^−7^), *Allisonella* (r = 0.8, q = 3.5 × 10^−7^), *Eubacterium coprostanoligenes group* (r = 0.7, q = 0.0002), *Howardella* (r = 0.8, q = 1.7 × 10^−7^), *Lachnospiraceae UCG-010* (r = 0.8, q = 6.9 × 10^−7^), *Mogibacterium* (r = 0.8, q = 4.0 × 10^−7^), *Olsenella* (r = 0.8, q = 1.8 × 10^−7^), *Sutterella* (r = 0.8, q = 2.1 × 10^−5^), uncultured *Lachnospiraceae* (r = 0.5, q = 0.03) and uncultured *Porphyromonadaceae* (r = 0.8, q = 6.5 × 10^−6^)) were positively correlated with faecal calprotectin. *Lachnospiraceae AC2044* group positively correlated with faecal M2-PK (r = 0.5, q = 0.04).

Correlations between predicted KEGG pathways and inflammatory markers (calprotectin (n = 27) and M2-PK (n = 26)) within CF subjects are presented in Supplementary Fig. 5. Six predicted pathways (benzoate degradation (KO362) (r = 0.6, q = 0.005), carbon fixation pathways in prokaryotes (KO720) (r = 0.5, q = 0.02), DDT degradation (KO351) (r = 0.6, q = 0.01), phenylalanine metabolism (KO360) (r = 0.6, q = 0.009), styrene degradation (KO643) (r = 0.5, q = 0.048) and tropane, piperidine and pyridine alkaloid biosynthesis (KO960) (r = 0.6, q = 0.007)) were positively correlated with faecal calprotectin. Indole alkaloid biosynthesis (KO901) (r = 0.5, q = 0.01) and Photosynthesis – antenna proteins (KO196) (r = 0.5, q = 0.04) positively correlated with faecal M2-PK.

### Associations with clinical measures in children with cystic fibrosis

The mean (SD) weight, height and BMI z-scores in children with CF were −0.0004 (1.1), 0.07 (1.2), and 0.1 (1.0) respectively. No significant correlations between anthropometric z-scores and alpha diversity indices (richness or Shannon index) were identified. Correlations between bacterial genera and anthropometric z-scores identified positive correlations between: (i) *Ruminococcaceae UCG* 014 and BMI (r = 0.6, q = 0.04), (ii) uncultured *VadinBE97* and weight (r = 0.5, q = 0.049), and (iii) uncultured *VadinBE97* and BMI (r = 0.6, q = 0.02). No significant correlations between anthropometric z-scores and predicted KEGG pathways were identified.

Lung function was recorded in 19 (70%) children, with a mean (SD) FEV1% of 98.9% (15.4). No significant correlations between FEV1% and alpha diversity indices (richness or Shannon index) were identified. FEV1% positively correlated with five genera: (i) *Adlercreutzia* (r = 0.6, q = 0.03), (ii) *Ruminococcaceae NK4A214* group (r = 0.6, q = 0.04), (iii) *Lachnospiraceae NC2004* group (r = 0.6, q = 0.04), (iv) *Tyzzerella* 3 (r = 0.6, q = 0.04), and (v) *Candidatus Soleaferrea* (r = 0.5, q = 0.047). No significant correlations between FEV1% and predicted KEGG pathways were identified.

### Metabolomics

Untargeted metabolomics data was available for 12 CF and 4 HC children as part of a previous study^22^. A total of 24,814 and 13,341 hits were detected in positive and negative ion modes, respectively, from all stool samples. Metabolites were identified using the human metabolome database (HMDB)^23^ and the mean (SD) number of identified metabolites per sample in positive and negative ion modes were 6,409 (690) and 1,845 (150), respectively. These lists of identified metabolites were used for all subsequent analyses. The metabolomes of CF and HC cohorts revealed distinct clustering (Supp. Figs. 7, 8). Principal component analysis (PCA) revealed a strong separation between CF and HC cohorts (Supp. Fig. 8A,C). A sensitivity analysis with 4 CF and 4 age and gender matched HC children also revealed a strong separation between CF and HC cohorts (Supp. Fig. 8B,D). Metabolites with a significantly different abundance between CF and HC cohorts (q < 0.05) are presented in a volcano plot (Supp. Fig. 9) and hierarchical clustering presented in Supplementary Fig. 10. Correlations between metabolites and bacterial genera (significant genera identified in Table 2) in children with CF are presented in Supplementary Fig. 10 and Supplementary Dataset 6. Correlations between metabolites and predicted KEGG pathways (significant pathways identified in Table 3) in children with CF are presented in Supplementary Fig. 12 and Supplementary Dataset 6.

A post hoc analysis of metabolites related to short-chain fatty acids (SCFA) was performed. A total of 25 and 2 hits were putatively identified as metabolites related to propanoate and/or butanoate metabolism^23^ in positive and negative ion modes, respectively. Two metabolites identified as butyric acid (positive ion mode): (i) *m/z* 106, RT 10.0 min and (ii) *m/z* 106, RT 9.8 min were constantly lower in children with CF compared to HC across all ages (normalised abundance estimate (SE) −10,134.4 (2,631.8), p = 0.002 and −6,841.5 (3,011.4), p = 0.04, respectively) (Supp. Fig. 13). One metabolite identified as pantetheine (negative ion mode; *m/z* 277, RT 11.5 min), an amide of pantothenic acid (vitamin B5), was significantly lower in children with CF than HC (normalised abundance median (IQR) 2,795 (1,343-16,628) vs. 35,418 (24,798-43,056), p = 0.045) (Supp. Fig. 14). Pantetheine is involved in the metabolism of propanoate to propionyl coenzyme A^23^.

## Discussion

We have demonstrated that the intestines of children with CF exhibit a marked taxonomic and inferred functional dysbiosis when compared to well-matched healthy controls. To our knowledge this is the first study to infer the function of intestinal bacterial communities in a paediatric CF population. We predicted an enrichment of genes involved in SCFA, antioxidant and nutrient metabolism, all of which are relevant to growth and nutrition in CF (Table 3; Fig. 4). We identified in a subset of samples, that children with CF have distinct metabolic profiles compared to HC. Reduced levels of butyric acid and pantetheine in the stool of children with CF provides support for the notion of increased SCFA (butanoate and propanoate) metabolism. Furthermore, the notion of pro-inflammatory GI microbiota in children with CF is supported by positive correlations between intestinal inflammatory markers and both genera and functional pathways. We also demonstrated an association between intestinal genera and both growth z-scores and FEV1%. Thus, this study provides evidence and insights into the links between alterations in the composition of the intestinal bacterial community with (i) predicted functional changes, (ii) intestinal inflammation, and (iii) clinical outcomes (e.g. growth and lung function). However, considering the nature of 16S rRNA-derived gene data, the precise pathogenic mechanisms in CF GI disease need further exploration and validation.

The paediatric CF GI microbiota demonstrated significantly lower α-diversity indices (richness and Shannon index) across all ages (Fig. 1). Increased GI diversity has been repeatedly associated with health, whilst decreased diversity has been associated with several inflammatory, metabolic, immune-mediated and systemic diseases^24^. These changes are clinically relevant in CF as the GI microbiota of young children has been proposed to be a determinant of respiratory and systemic disease progression^25^. Intestinal dysbiosis is further supported by the significant difference in bacterial communities between CF and HC cohorts using both weight and unweighted UniFrac distances (Fig. 2). Similarly Vernocchi *et al*.^9^ demonstrated that children (aged 1 to 6 years) with CF have lower alpha diversity (Chao1) and a distinct beta diversity (unweighted UniFrac distance) compared to HC.

Similar to previous reports, we identified a marked increase in the relative abundance of *Proteobacteria* (genera *Escherichia*, *Shigella*, *Enterobacter* and *Morganella*), particularly in the first few years of life (Fig. 3; Table 2 and Supp. Figure 3A,E)^6,8,26^. In our samples, *Escherichia* was predominantly comprised of *Escherichia coli* species, which have been shown to positively correlate with faecal measures of nutrient malabsorption and inflammation in CF^26^. The relative abundances of functions related to two intestinal pathogens were increased in CF, shigellosis (p = 3.0 × 10^−8^) and Vibrio cholerae (pathogenic cycle) (p = 0.0001) (Table 3). This likely reflects the enrichment of *Escherichia* and *Enterobacter*, which share several common genes, rather than the presence of the pathogens themselves. The clinical implications of this however are unclear.

The increased relative abundance of *Veillonella* in CF samples (Fig. 3; Table 2 and Supp. Figure 3E) has also previously been observed^8,25,27^ and *Veillonella* comprises part of the core respiratory microbiota in CF^25,27^. *Veillonella* is known to produce propionate (i.e. propanoate) from lactate fermentation^28^ and the relative abundance of airway *Veillonella* has been reported to inversely correlate with airway inflammation^29^.

*Fusobacterium* was significantly enriched in children with CF compared to HC, and this genus has been extensively linked to colorectal cancer^30^. This finding is relevant given the 5–10 times greater risk of colorectal cancer in adults with CF compared to the general population^31^.

Several members of the *Ruminococcaceae* family were reduced in CF samples (Fig. 3; Table 2 and Supp. Figure 3D,E) and they are known producers of SCFAs (from fermentation of carbohydrates, including resistant starch)^32^. Interestingly in CF children, the relative abundance of *Ruminococcaceae UCG* 014 positively correlated with BMI z-scores (r = 0.6, q = 0.04) and *Ruminococcaceae NK4A214* group positively correlated with FEV1% (r = 0.6, q = 0.04). Dietary studies examining resistant starch intake and potential interventional studies may be worth exploring in children with CF.

The relative abundance of *Alistipes* was decreased in CF samples (Fig. 3; Table 2 and Supp. Figure 3E) and species of this genus are known to produce succinic acid^33^. In mice, dietary succinate was identified as a substrate for intestinal gluconeogenesis and improved glucose homeostasis^34^. This may be relevant to the exploration of factors associated with glucose abnormalities in children with CF, an increasingly recognised problem^35^.

Intestinal inflammation as measured by faecal calprotectin is clinically relevant given its negative correlation with height and weight z-scores in CF children^11^. Both faecal calprotectin and M2-PK were significantly elevated in our CF cohort compared with HC (Fig. 5). Interestingly, *Acidaminococcus* showed a strong positive correlation with calprotectin (r = 0.83, q = 3.9 × 10^−7^) in CF (Supp. Figure 4D). Increased *Acidaminococcus* relative abundance has been associated with lower future height z scores in twin-cohorts of children from Malawi and Bangladesh^36^. *Acidaminococcus sp*. consume glutamate (as their sole source of carbon and energy) which is important to gut amino acid metabolism, nitrogen balance, barrier function and epithelial restitution^36^.

Children with CF appeared to have distinct metabolic profiles compared to HC (Supp. Figs. 7–10). Interestingly, those children with CF and PS (samples CF33 and CF36) appeared similar to HC rather than PI CF children (Supp. Figs. 7, 8, 10A), providing support for a gradation effect with the degree of CFTR dysfunction. The taxonomic and functional dysbiosis which we have identified, along with the known CFTR dysfunction are plausible factors contributing to the altered metabolic profiles in children with CF (compared to HC). In children with CF, we identified several significant correlations between metabolites and (i) bacterial genera (Supp. Fig. 11), and (ii) predicted KEGG pathways (Supp. Fig. 12), suggesting that intestinal dysbiosis has an influence on metabolic profiles.

In our CF cohort there was a predicted enrichment of genes involved in the metabolism of SCFAs propanoate, butyrate and a precursor, pyruvate (Table 3 and Fig. 4A,B). Short-chain fatty acids have several beneficial effects on GI tract health including, improved intestinal motility, reduced intestinal inflammation, promotion of differentiation of regulatory T cells and regulation of both fatty acid and glucose metabolism^37,38^. Recently, Vernocchi *et al*.^9^ reported butyrate and propionate levels to be lower in children with CF compared to HC. Similarly, in a subset of samples, we identified lower levels of butyric acid and pantetheine in children with CF compared to HC. It is likely that in children with CF, intestinal microbiota have an increased propensity for the metabolism of beneficial SCFAs. The variable trend of propanoate metabolism with advancing age in CF subjects (Fig. 4A) highlights the need to perform a quantitative analysis of dietary-fibre intake and faecal excretion of SCFAs.

In children with CF, we predicted an enrichment of bacterial genes associated with glutathione, taurine and hypotaurine metabolism and ubiquinone (coenzyme Q10) biosynthesis (Table 3 and Fig. 4G–I). Both glutathione and taurine are antioxidants which are protective against oxidative stress and exhibit anti-inflammatory properties^39^. This is notable in light of a recent randomised controlled trial on oral reduced glutathione demonstrating improved growth z-scores and decreased faecal calprotectin in CF children^18^. Antioxidants have been explored as a therapeutic intervention in CF lung disease, however evidence is lacking to support their use^40^. Little is known about the role of oxidative stress in the intestinal metabolism of CF and further exploration into the role of antioxidants for CF GI disease is warranted.

The relative abundance of predicted functions associated with phenylalanine metabolism were increased in CF (p = 0.0002) and also positively correlated with calprotectin (r = 0.6, q < 0.01) (Table 3, Fig. 4J and Supp. Fig. 14). Phenylalanine is an essential and aromatic amino acid which may play several key roles including: (i) attenuating intestinal inflammation through activating calcium-sensing receptors (in piglets)^41^; (ii) inhibiting TNF-α production^42^; and (iii) enhancing immune responses^42^. Quantification of phenylalanine intake may be a useful measure in future CF studies.

In our CF cohort predicted functions related to the metabolism of thiamine (vitamin B_1_) (p = 1.3 × 10^−6^) and biosynthesis of pantothenate (vitamin B_5_) and coenzyme A (CoA) (p = 3.1 × 10^−5^) were decreased (Table 3 and Fig. 4K,L). These two water-soluble vitamins help metabolise carbohydrates, fats and proteins and their depletion may provide insights into the pathophysiology of malnutrition in CF (despite pancreatic enzyme replacement therapy, high-fat high-calorie diets and vitamin supplementation^43^).

Some limitations of this current study need to be considered. Firstly, functional pathways were inferred from 16S rRNA gene data and not measured directly. However, given that 84.6% of our 16S rRNA genes could be assigned a functional profile, we believe our analysis still has strong predictive power. Secondly, although our sample size is small, it is still the largest study to date analysing intestinal microbiota function in CF children and HC (age and gender matched) and exceeded the minimum number required (calculation below). Given only three CF children were PS, we were unable to make any meaningful comparisons with PI children. Furthermore, for children with CF, FEV1% was not recorded at the time of stool sampling but within the preceding 12 months. And finally, confounding factors, including antibiotic usage and altered dietary regimes between CF and HC, were not controlled for with this study. A quantitative analysis of dietary intake and direct measures of faecal proteins and metabolites (i.e. targeted metabolomics for measurement of SCFA levels) would be required to validate the findings presented in this study. Although our comparison of metabolomes between CF and HC cohorts was unmatched, we performed a sensitivity analysis with 4 CF and 4 age and gender matched HC which yielded similar results (Supp. Fig. 8B,D).

In conclusion, there exists both a taxonomic and inferred functional dysbiosis, which provides insights into paediatric CF GI disease. Future observational or interventional studies should simultaneously evaluate dietary intake, abdominal symptoms and faeces. Further exploration of potential CF GI therapeutics including antioxidants (e.g. glutathione), SCFAs (e.g propanoate and butyrate), amino acids (e.g phenylalanine) and gut microbiota modulators (e.g. prebiotics including resistant starch, probiotics and synbiotics) is warranted.

## Methods

### Study population

We performed a prospective, cross-sectional, observational study in children with CF and HC. The subjects and samples for this analysis were collected as part of three prior studies evaluating the progression of intestinal microbiota^6^ and inflammation in children with CF^12,14^. Children with CF and HC (aged 0 to 18 years) were prospectively recruited from the outpatient clinics (CF and Orthopaedic/Plastics clinics respectively) at Sydney Children’s Hospital Randwick, Australia. In addition, inclusion and exclusion criteria were as follows:

Inclusion criteria:

- Children with CF diagnosed according to the United States Cystic Fibrosis Foundation consensus criteria^44^.
- Exocrine pancreatic insufficiency (PI) or pancreatic sufficiency (PS) defined based on the 72-hour faecal fat and/or faecal elastase-1^45,46^.
- Children with CF on oral or inhaled antibiotic therapy were not excluded.
- Healthy controls included children without CF or any gastrointestinal disease.

Exclusion criteria:

- Patients requiring antibiotic therapy for a pulmonary exacerbation^47^ or intravenous antibiotics in the preceding four weeks before sampling.
- Any child (CF or HC) with gastroenteritis, on oral corticosteroids, probiotics and/or non-steroidal anti-inflammatory drugs in the preceding two weeks before sampling.

Children with CF were matched to healthy controls for gender and age (as closely as possible).

### Sample collection and processing

A single stool sample from each subject was collected and stored immediately at −80 °C, or stored at −20 °C (home freezer) until transport to the laboratory for storage at −80 °C. Thawing of the sample during transport did not occur^6^. At the time of sample collection, demographic and anthropometric (height, weight and body mass index (BMI) z-scores) data was recorded. In CF children older than four years, the forced expiratory volume in one second, percent predicted (FEV1%) from their most recent lung function test was recorded (within the preceding 12 months).

DNA was extracted from stool samples and sequenced as previously described^6^. The 16S rRNA genes of the gut microbiota were amplified with primers 27F (AGAGTTTGATCMTGGCTCAG) and 519R (GWATTACCGCGGCKGCTG) spanning the V1–V3 regions and sequenced using the Illumina MiSeq platform (v3, 2 × 300 bp).

### Processing of 16S rRNA data

Paired-end sequences were quality filtered using Trimmomatic v0.36^48^: low quality reads were truncated if the quality dropped below 15 in a sliding window of 4 bp. Reads shorter than 36 bp were removed Remaining paired-end reads were merged and then filtered using USEARCH v9.2.64^49^. Filtering included the removal of reads shorter than 400 bp or longer than 500 bp as well as the removal of low-quality reads (expected error > 1) and reads with more than one ambiguous base. Processed sequences of all samples were concatenated into one file and subsequently dereplicated into unique sequences. These sequences were clustered in unique sequences (zero-distance operational taxonomic unit; zOTU) with the unoise2^50^ algorithm implemented in USEARCH. A denovo chimera removal step was included in the unoise step. Subsequently, the uchime algorithm was utilized in reference mode with the SILVA 123 database^51^ to remove remaining chimeric sequences. Chimera-free sequences were classified by BLASTn alignment against the SILVA database. Concatenated sequences of all sequences were mapped on the final set of zOTUs to calculate the abundance of each zOTU in any given sample. All non-bacterial sequences were removed based on their taxonomic classification. A phylogenetic tree was constructed using MUSCLE v3.8.31^52^.

Samples with <22,578 sequences per sample were removed, with this threshold being based on a trade-off between sequencing depth and the number of samples for paired analysis. Coverage per sample was estimated with a Michaelis-Menten Fit.

Functional profiles were inferred from 16S rRNA data using Tax4Fun^53^. Tax4Fun predicts the functional capabilities of the microbial communities by mapping the 16S rRNA gene sequences with the functional annotation of sequenced prokaryotic genomes in the Kyoto Encyclopedia of Genes and Genomes (KEGG).

### Inflammatory biomarkers

Calprotectin was extracted and measured as described in a previous study^12^ using the PhiCal kit (Calpro, San Diego, CA, US). The lower limit of detection for the assay was 19.5 mg/kg. Calprotectin > 50 mg/kg is considered elevated. Faecal M2-PK was extracted and measured as described in a previous study^14^ using the ScheBo Tumour M2-PK kit (ScheBo Biotech, Giessen, Germany). The lower limit of detection of the assay was 1 U/mL.

### Stool metabolomics

A subset of samples underwent untargeted ultra-high performance liquid chromatography-tandem mass spectrometry (UHPLC-MS/MS) as previously reported, with the primary aim of exploring inflammatory pathways^22^. Metabolites were identified using the human metabolome database (HMDB)^23^ and the normalised abundances of these identified metabolites were uploaded into Perseus v1.6.6.0. The normalised abundances were then log(2) transformed. Metabolite rows were filtered for at least 70% valid values in at least one group. Missing values were replaced from a normal distribution (total matrix). Proteins with a significantly different abundance between CF and HC cohorts were determined using a two sample Student’s T-test (permutation-based FDR < 0.05). The normalised abundances were then Z-score normalised which were used for downstream analysis. A post hoc analysis of metabolites related to propanoate and butanoate metabolism (Supp. Dataset 7)^23^ was also performed.

### Sample size

The minimum required sample size for this study was calculated using GLIMMPSE 2.0.0 (https://glimmpse.samplesizeshop.org/#/) and based on the age-adjusted mean (95% CI) Shannon index reported in our initial gut microbiota study for CF and HC cohorts (2.75 (2.51–2.98) and 3.90 (3.72–4.08), respectively)^6^. A minimum sample size of 10 subjects was required to reject the null hypothesis that the population means of the CF and HC groups are equal with probability (power) 0.8 and Type I error of 0.05.

### Statistical analysis

Statistical analysis was performed in R v3.4.4. Alpha and phylogeny-based beta diversity indices were calculated with a dataset random subsampled to 22,578 sequences per sample. Pairwise weighted and unweighted UniFrac distances between samples^54^ were calculated using the GUniFrac package^55^ and used to generate non-metric multidimensional scaling (NMDS) plots. Continuous variables were compared using a paired t-test or Wilcoxon signed-rank test for parametric and non-parametric data, respectively (p < 0.05 considered statistically significant). Generalised linear models (glm function; using a gaussian distribution) were constructed to control for age when comparing continuous variables between pairs. Permutational multivariate analysis of variance (PERMANOVA) tests (permutations = 1000) were utilised to test if beta diversity significantly differed between groups (‘CF vs HC’ and ‘male vs female’) and for age using the vegan function adonis^56^. Graphs were generated using ggplot2 in R^57^. A significant difference in abundance of taxa or zOTU between CF and HC groups was assessed using the ANCOM package v1.1-3 (Benjamini & Hochberg correction for multiple comparisons, q < 0.05)^58^. A significant difference in abundance of functional pathways was assessed with multiple Wilcoxon signed-rank tests corrected for multiple testing using a Dunn-Sidak correction (n = 275, p < 0.00019). Correlations between continuous variables were assessed using Pearson or Spearman correlations (Benjamini & Hochberg correction, q < 0.05)^59^.

### Patient consent

Written informed consent was obtained from each subject or caregiver(s) and the study was carried out in accordance with the approved guidelines.

### Ethics approval

This study was approved by the South Eastern Sydney Area Health Service, Human Research Ethics Committee, Sydney Australia (HREC ref no: 10/240).

### Data sharing statement

The datasets generated and analysed during the current study are available upon reasonable request to the corresponding author.

## Supplementary information

Supplementary Material

Supplementary Dataset 1

Supplementary Dataset 2

Supplementary Dataset 3

Supplementary Dataset 4

Supplementary Dataset 5

Supplementary Dataset 6

Supplementary Dataset 7

Supplementary Dataset 8

Supplementary Dataset 9
