# Supplementary material for: Gut Microbiota in Children With Cystic Fibrosis: A Taxonomic and Functional Dysbiosis
Source: Sci Rep. 2019 Dec 9;9:18593. doi: 10.1038/s41598-019-55028-7 (PMC6901462; doi:10.1038/s41598-019-55028-7)
Supplement: Supplementary file 1 — Supplementary Material [file 41598_2019_55028_MOESM1_ESM.pdf]

**SUPPLEMENTARY MATERIAL**

# GUT MICROBIOTA IN CHILDREN WITH CYSTIC FIBROSIS: A TAXONOMIC AND FUNCTIONAL DYSBIOSIS


**Authors**

Michael J. Coffey,[1] Shaun Nielsen,[2] Bernd Wemheuer,[2] Nadeem O. Kaakoush,[3] Millie Garg,[1] Bronwen Needham,[2] Russell Pickford,[4] Adam Jaffe,[1,5,6] Torsten Thomas[2] & *Chee Y. Ooi.[1,5,7]

[1]Discipline of Paediatrics, School of Women’s and Children's Health, University of New South Wales, Sydney NSW, Australia.

[2]Centre for Marine Science and Innovation, School of Biological, Earth and Environmental Sciences, University of New South Wales, Sydney NSW, Australia.

[3]School of Medical Sciences, University of New South Wales, Sydney NSW, Australia.

[4]Bioanalytical Mass Spectrometry Facility, Mark Wainwright Analytical Centre (MWAC), University of New South Wales, Sydney NSW, Australia.

[5]Molecular and Integrative Cystic Fibrosis (miCF) Research Centre, High Street, Randwick NSW, Australia.

[6]Department of Respiratory, Sydney Children's Hospital, High Street, Randwick NSW, Australia.

[7]Department of Gastroenterology, Sydney Children's Hospital, High Street, Randwick NSW, Australia.

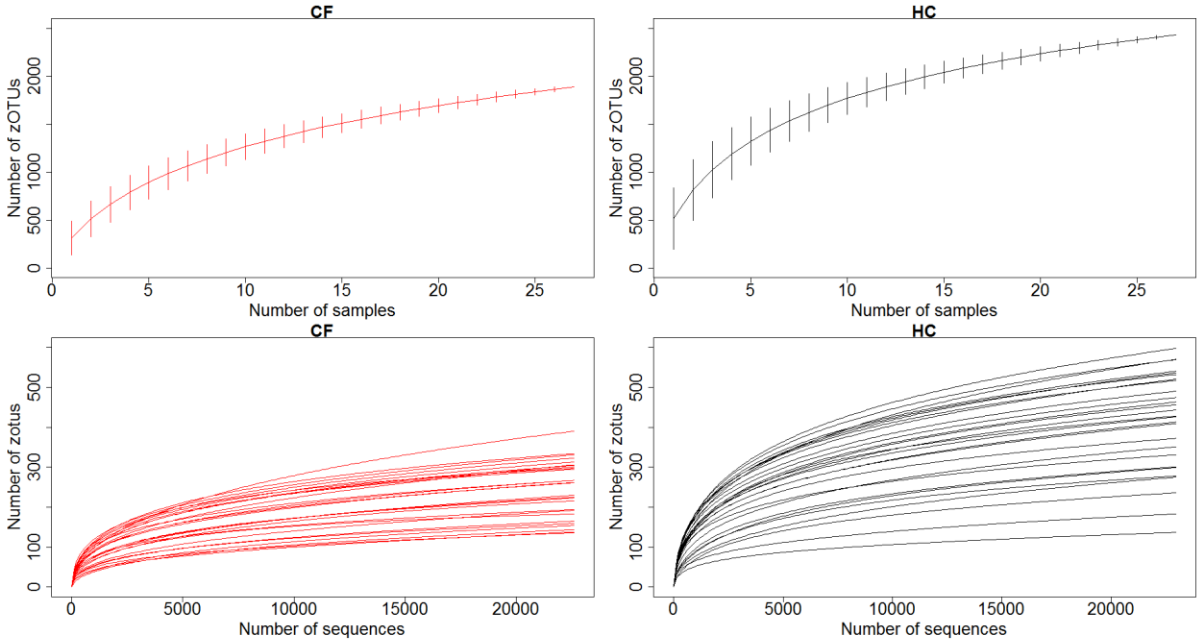


**Supplementary Figure 1**. Rarefaction curves for CF and HC cohorts. The number of zOTUs given the number of samples (top row) and the number of 16S rRNA gene sequences per stool sample (bottom row)

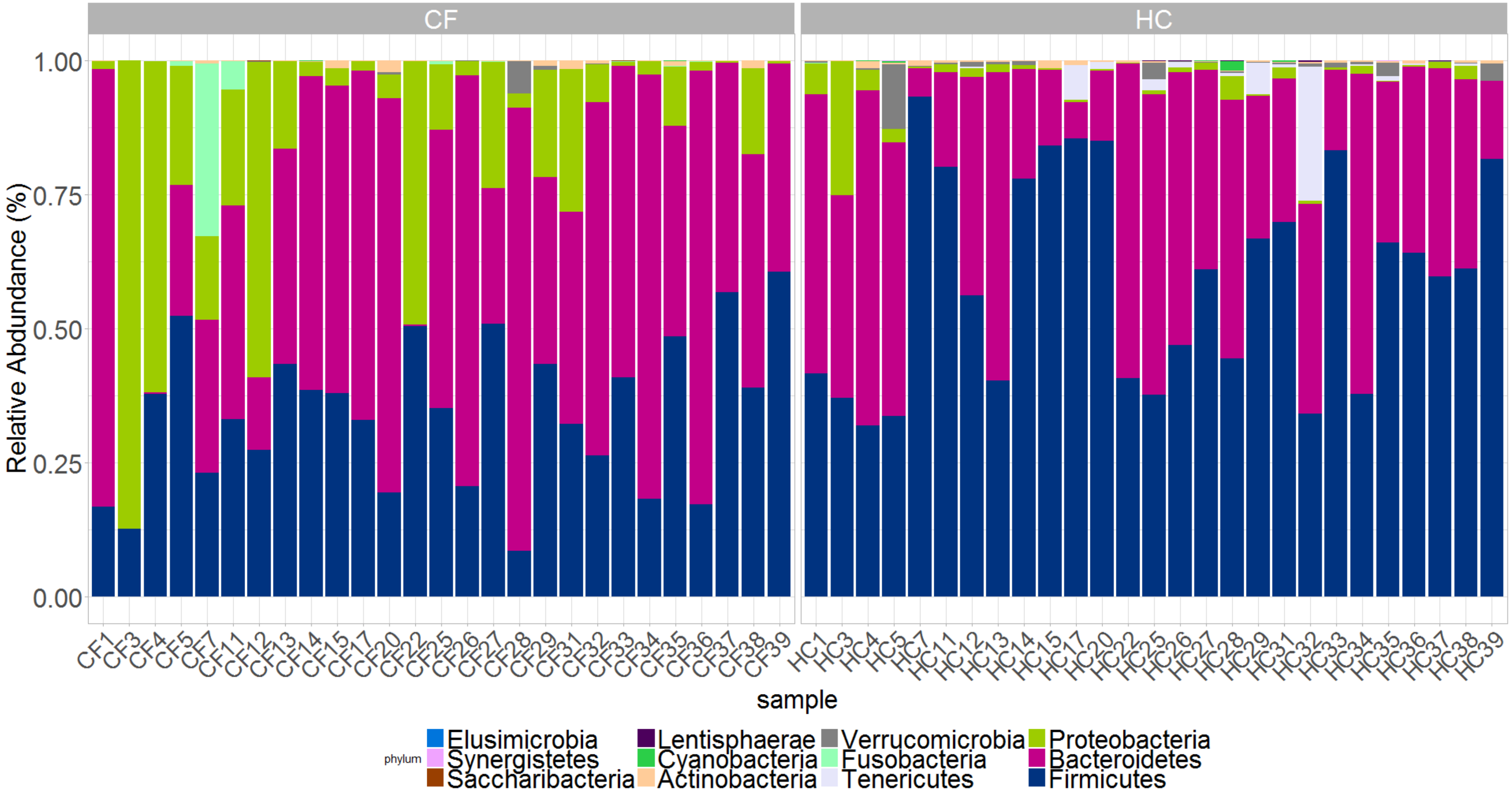


**Supplementary Figure 2.** Relative abundance of all bacterial phyla for CF and HC subjects. Samples ordered in increasing age (from left to right).

## A. Phylum

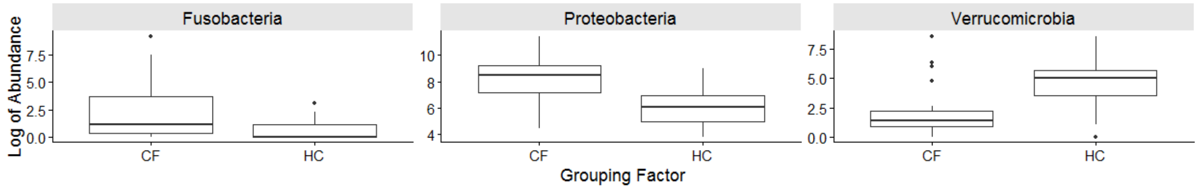


## B. Class

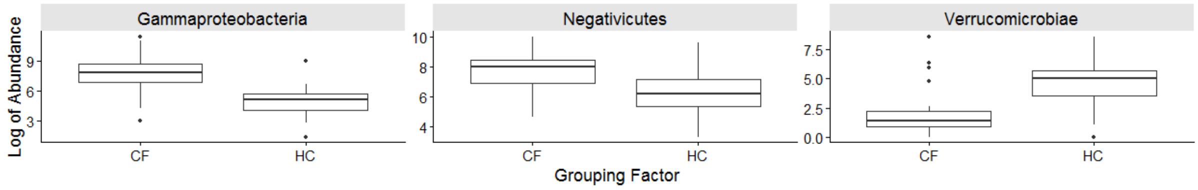


## C. Order

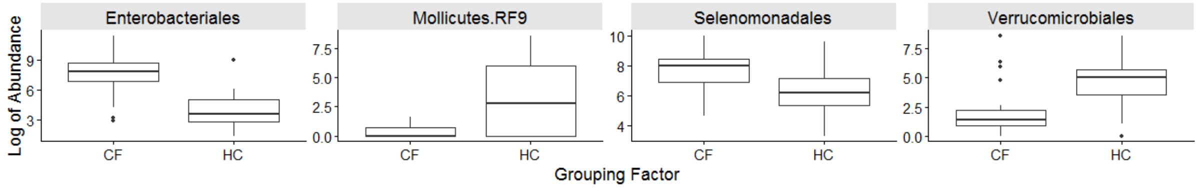


## D. Family

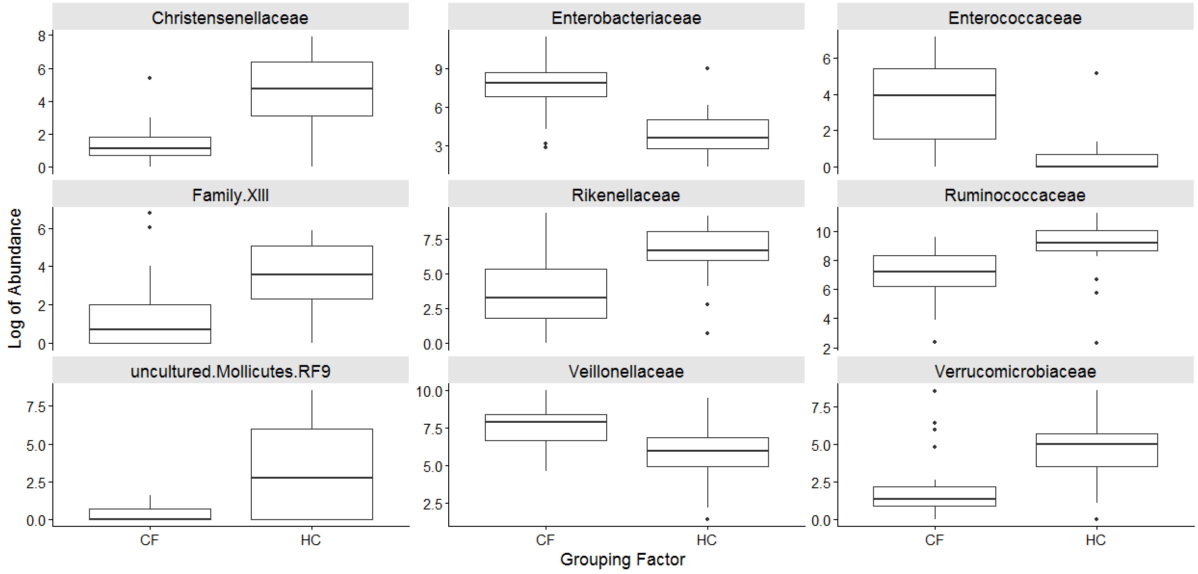

**E. Genus**

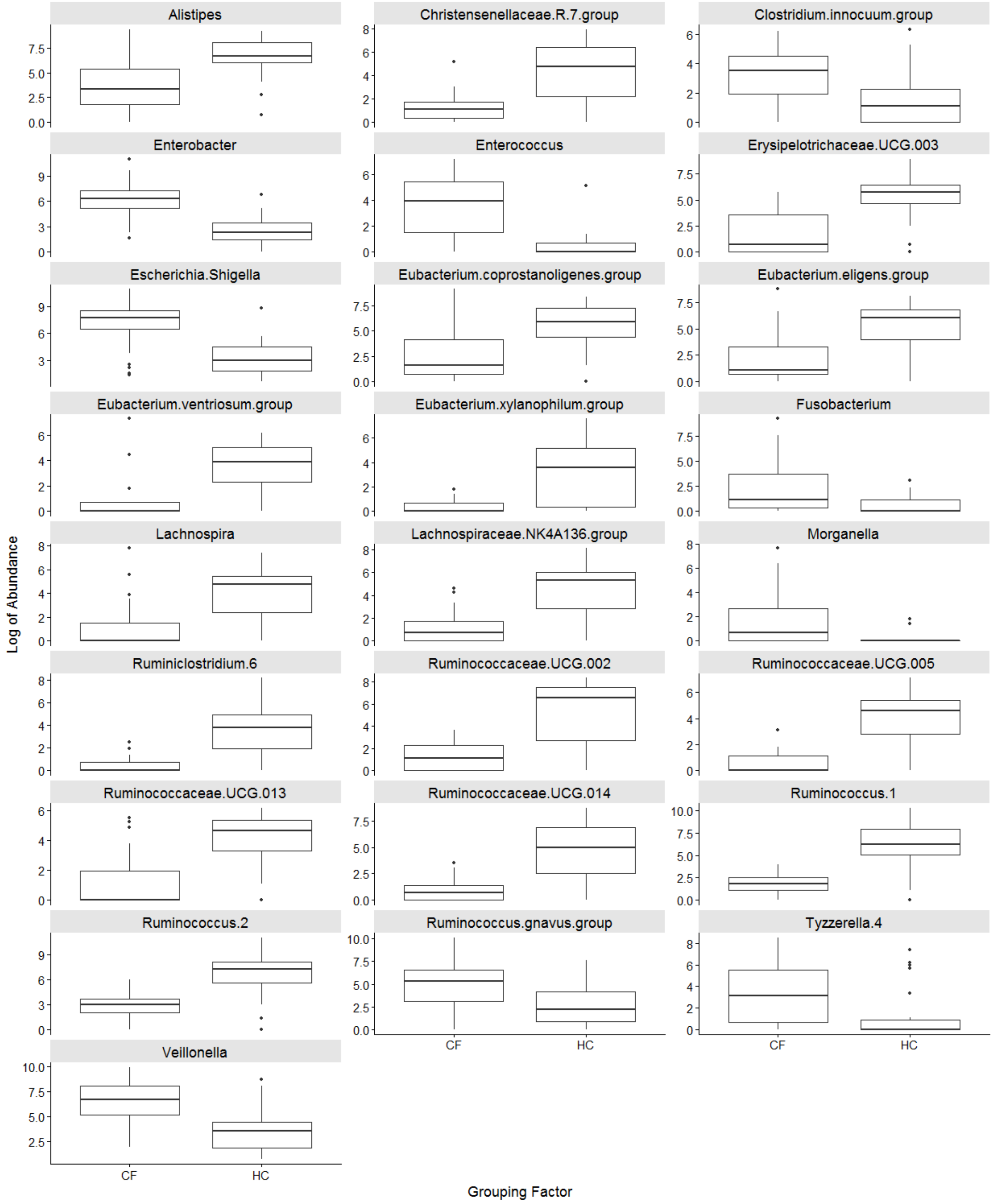


**Supplementary Figure 3.** Bacterial taxa with a significantly different abundance between CF and HC cohorts (at each taxonomic rank) using ANCOM analysis (q<0.05).

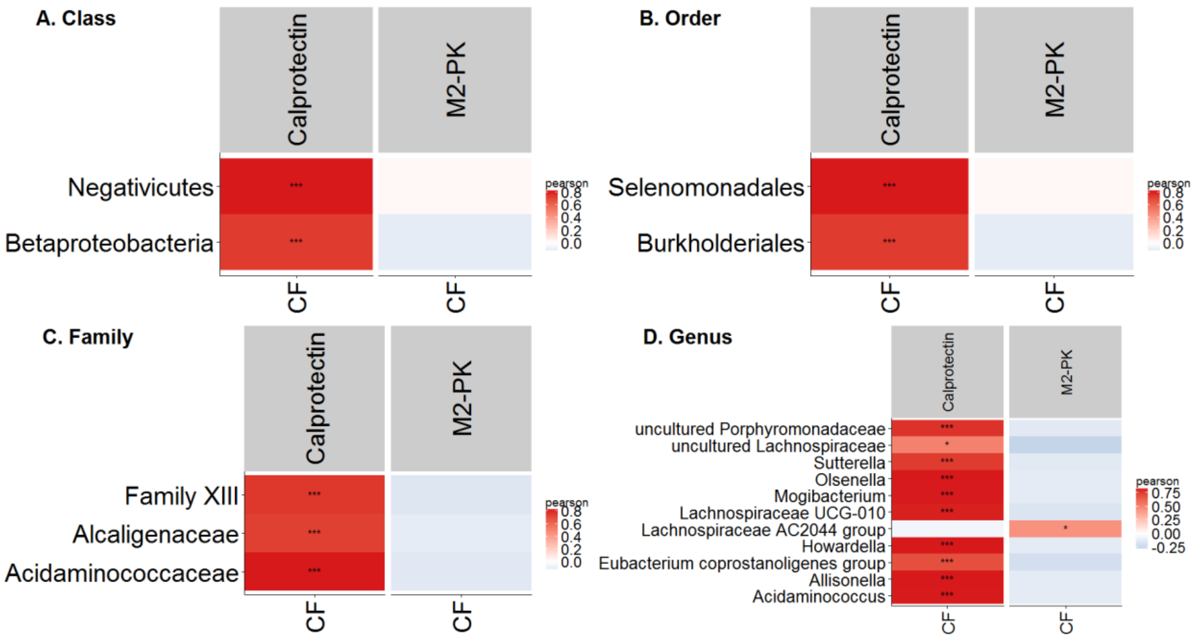


**Supplementary Figure 4**. Correlations between bacterial taxa and inflammatory markers (calprotectin (n=27) and M2-PK (n=26)) within CF subjects (at each taxonomic rank; no significant correlations at the phylum level). Pearson correlations corrected for multiple testing. *, q<0.05; **, q<0.01; ***, q<0.001.

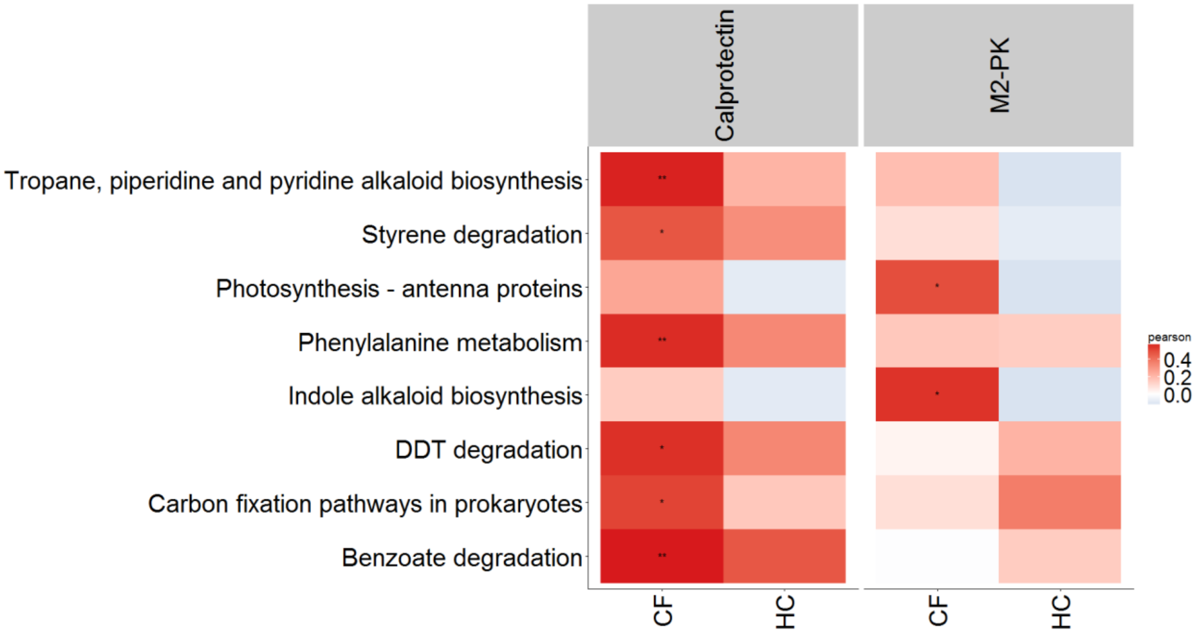


**Supplementary Figure 5**. Correlations between KEGG pathways and inflammatory markers (calprotectin (n=27) and M2-PK (n=26)) within CF subjects. Pearson correlations corrected for multiple testing. *, q<0.05; **, q<0.01; ***, q<0.001.

**A. Positive Ion Mode**

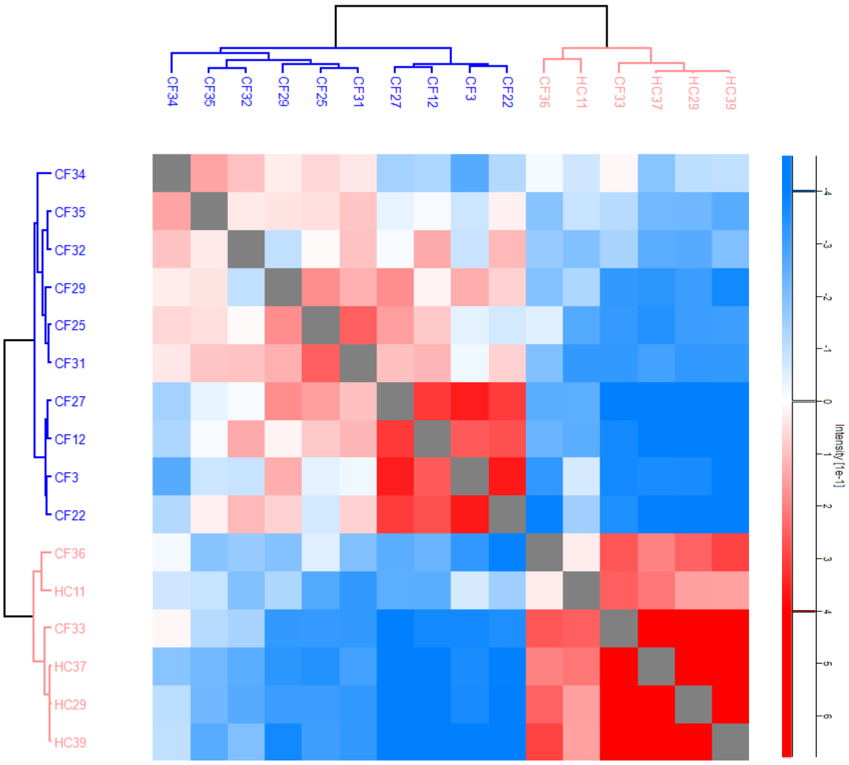


**B. Negative Ion Mode**

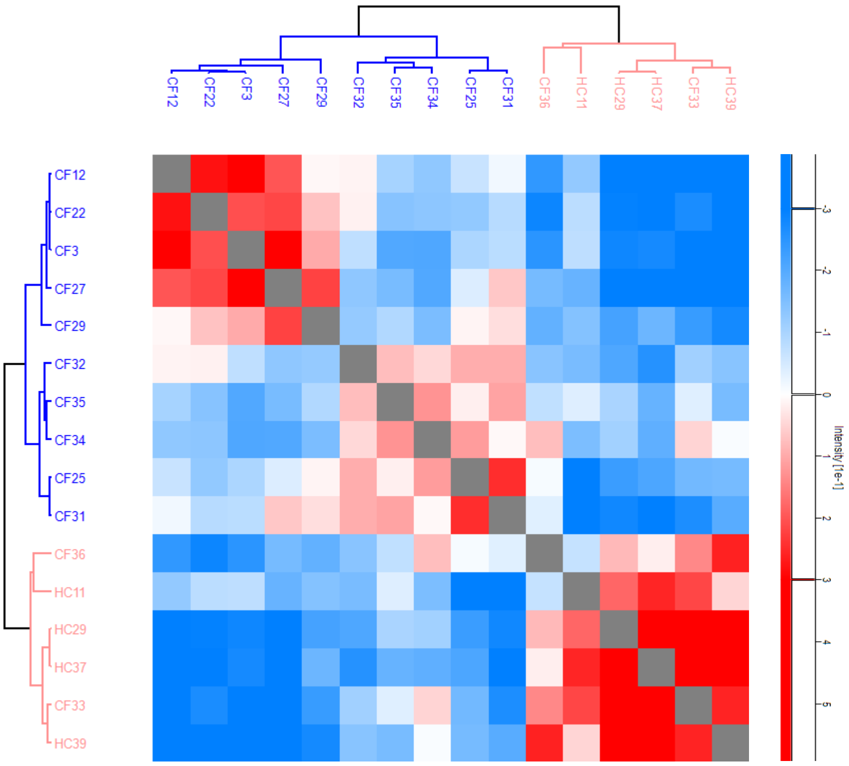


**Supplementary Figure 7**. Hierarchical clustering of Pearson correlation coefficients of normalised abundances in positive (**A**) and negative (**B**) ion modes. Metabolites identified in at least 70% of at least 1 group (CF or HC). CF33 and CF36 are PS and cluster with HC subjects.

**A. Positive Ion Mode**

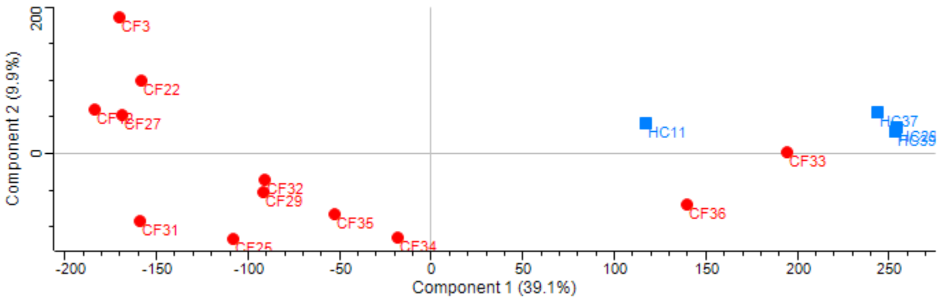


**B.**

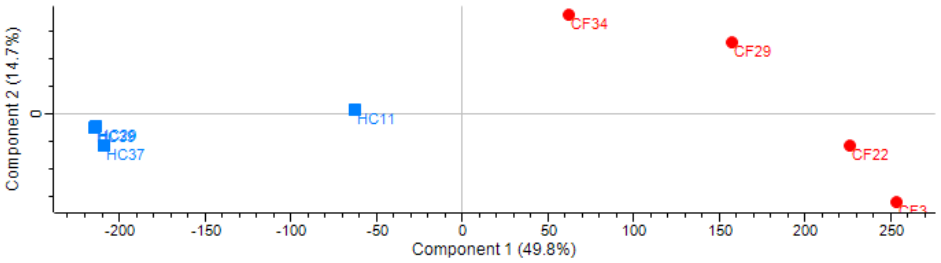


**C. Negative Ion Mode**

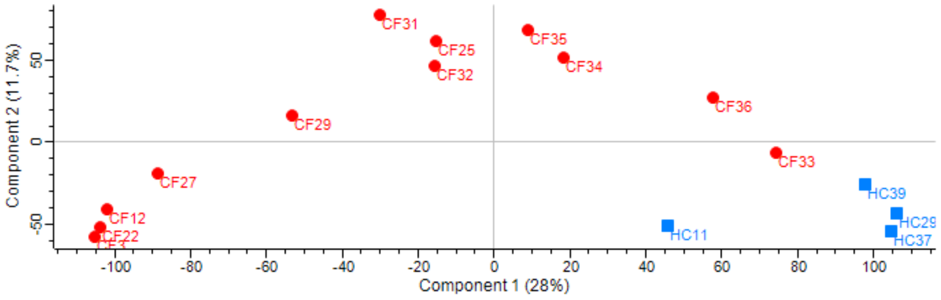


**D.**

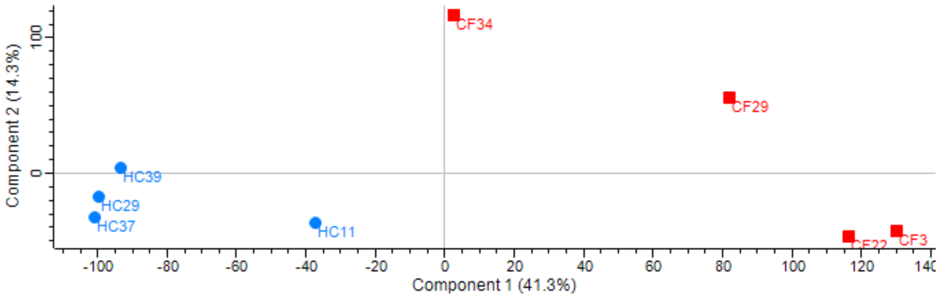


**Supplementary Figure 8**. Principal component analysis (PCA) plots of normalised abundances in positive (**A,B**) and negative (**C,D**) ion modes. PCA plots of all 12 CF and 4 HC subjects (**A,C**). Sensitivity analysis with PCA plots of 4 CF and 4 HC (age and gender matched) subjects (**B,D**). Metabolites identified in at least 70% of at least 1 group (CF or HC). CF33 and CF36 are PS and cluster closer to HC than CF subjects. Red, CF samples. Blue, HC samples.

**A. Positive Ion Mode**

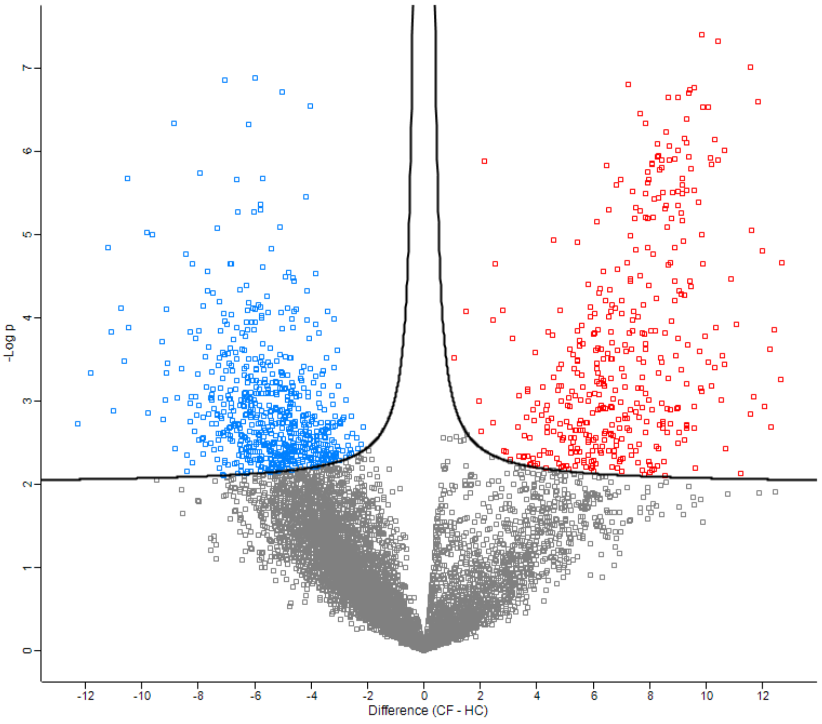


**B. Negative Ion Mode**

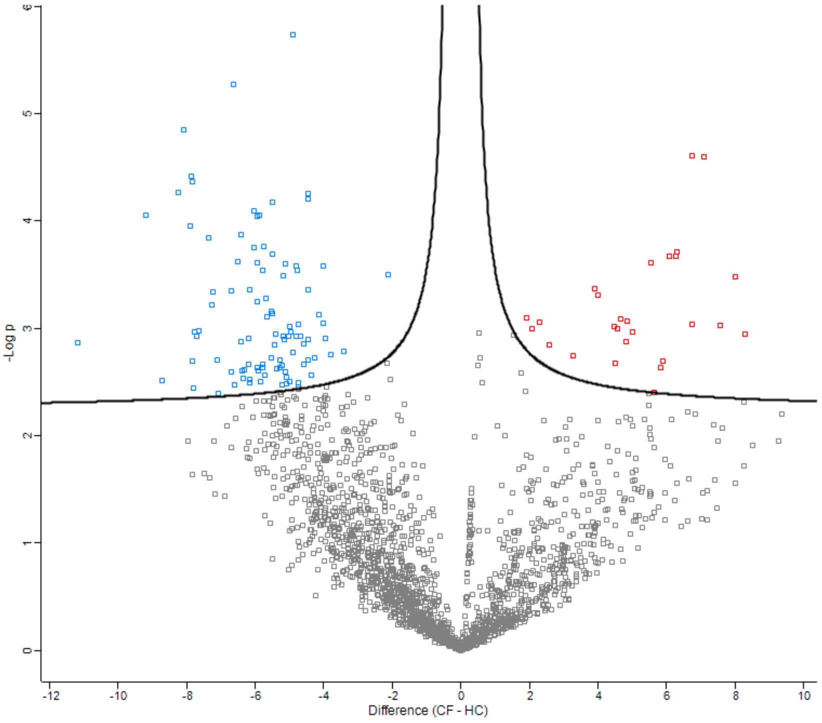


**Supplementary Figure 9**. Differentially expressed metabolites in positive (**A**) and negative (**B**) ion modes. Volcano plots using a t-test of normalised abundances between CF and HC cohorts (FDR<0.05). Metabolites identified in at least 70% of at least 1 group (CF or HC). Red, metabolites significantly upregulated in CF samples. Blue, metabolites significantly downregulated in CF samples.

**A. Positive Ion Mode**

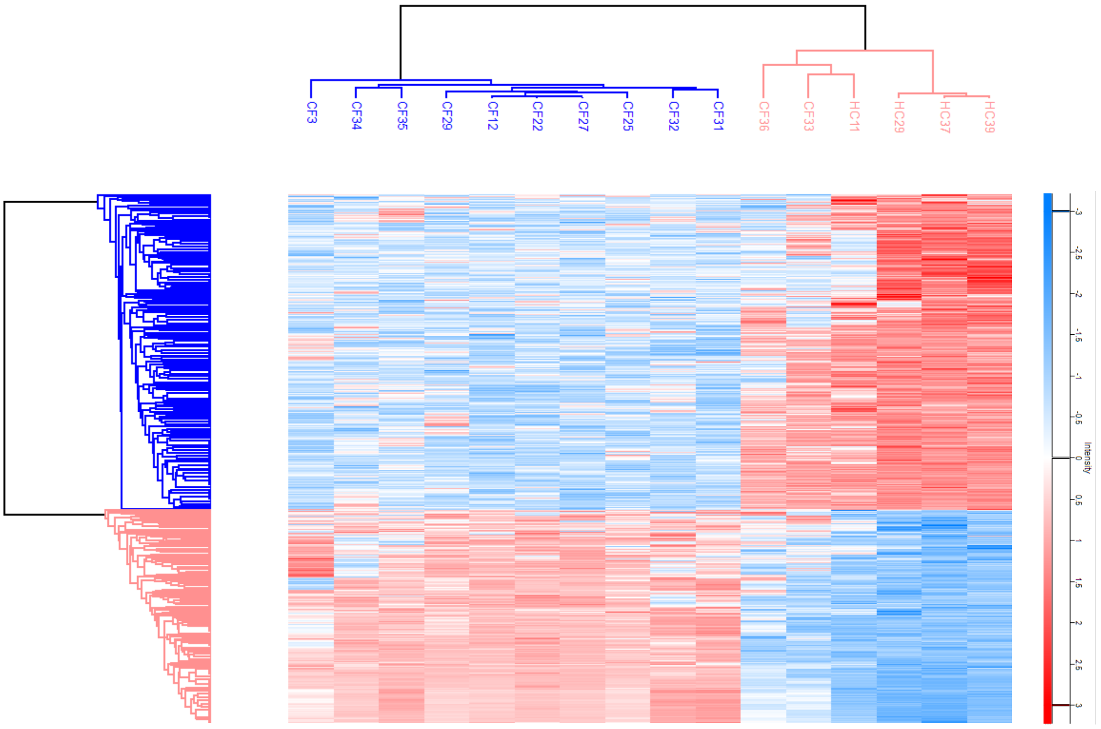


**B. Negative Ion Mode**

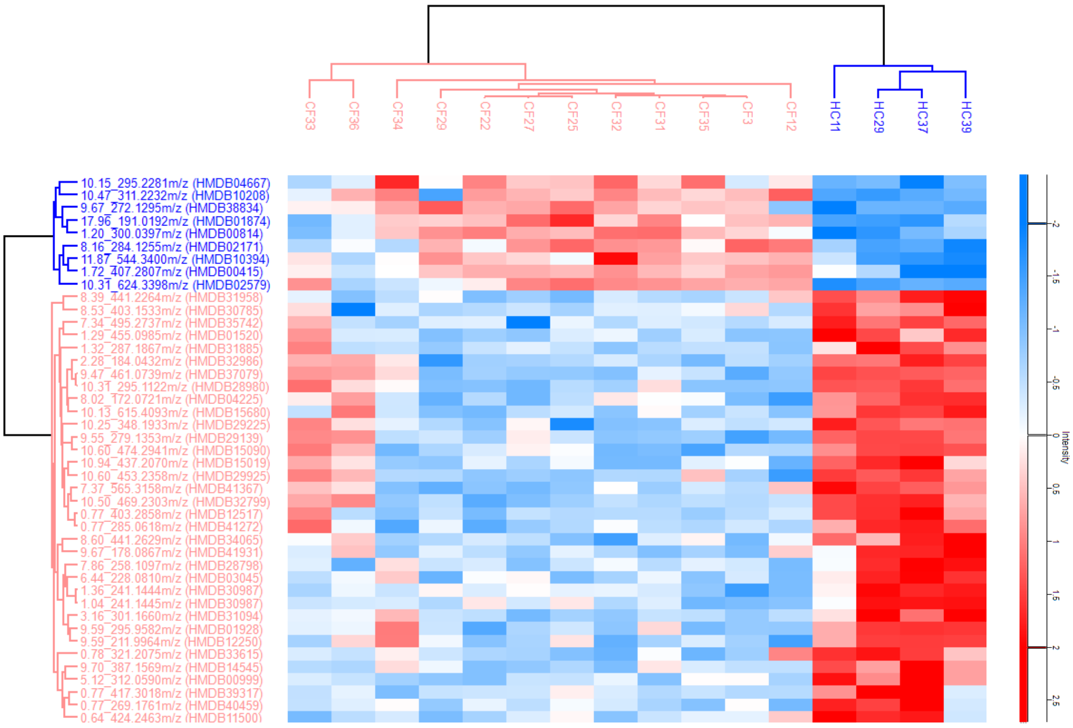


**Supplementary Figure 10**. Hierarchical clustering of differentially expressed metabolites. The normalised abundance of 928 metabolites were significantly different between CF and HC cohorts in positive ion mode (**A**) (FDR<0.05). The normalised abundance of 43 metabolites were significantly different between CF and HC cohorts in negative ion mode (**B**) (FDR<0.05). CF33 and CF 36 are PS and cluster with HC subjects in positive ion mode results.

## A. Positive Ion Mode

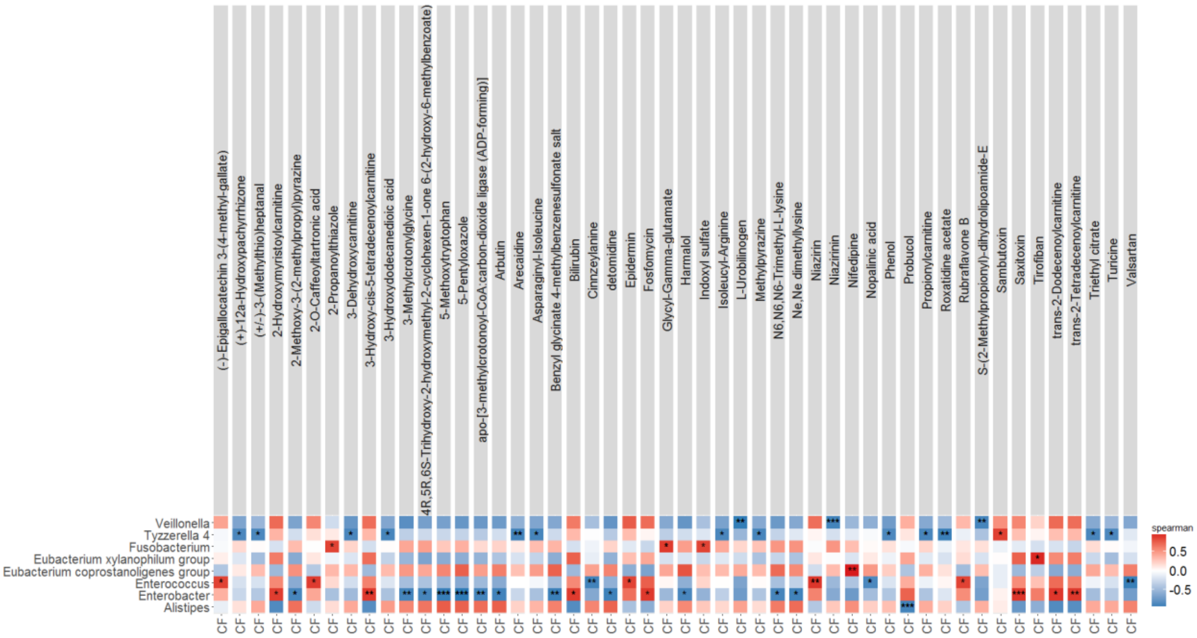


## B. Negative Ion Mode

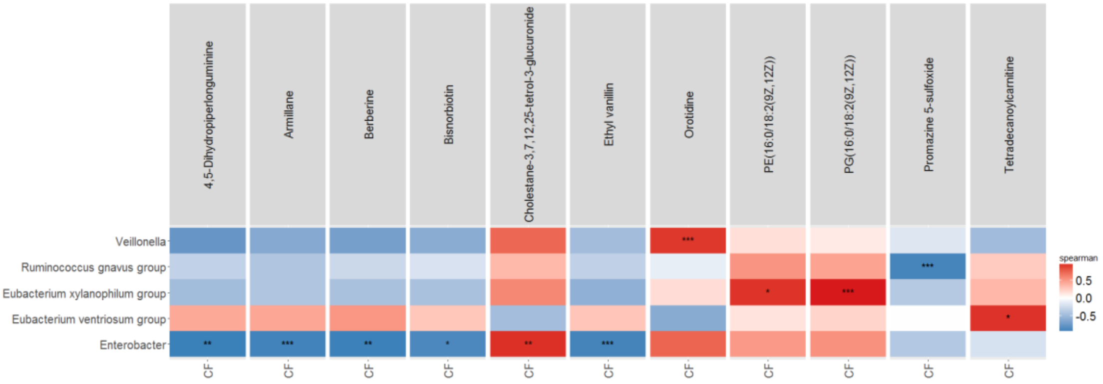


**Supplementary Figure 11**. Correlations between the normalised abundances of metabolites in positive ion mode (**A**) or negative ion mode (**B**), and the relative abundances of bacterial genera (significant genera identified in Manuscript Table 2) in children with CF. Spearman correlations corrected for multiple testing (B-H correction). *, q<0.05; **, q<0.01; ***, q<0.001.

## A. Positive Ion Mode

### (i) Glutathione metabolism

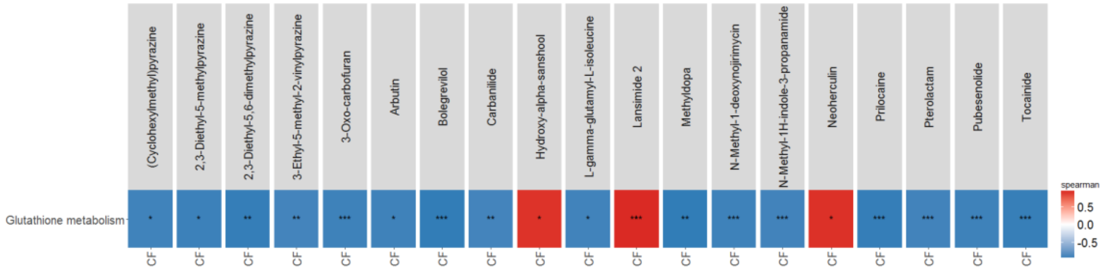


### (ii) Taurine and hypotaurine metabolism

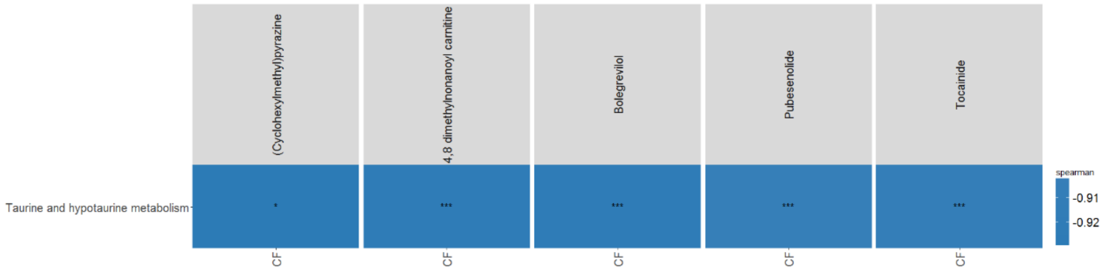


### (iiI) Propanoate metabolism

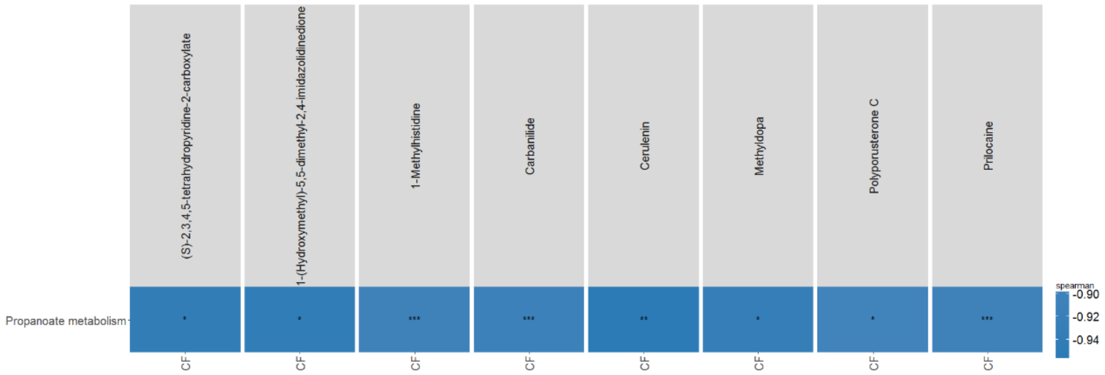


### (iv) Biosynthesis of unsaturated fatty acids

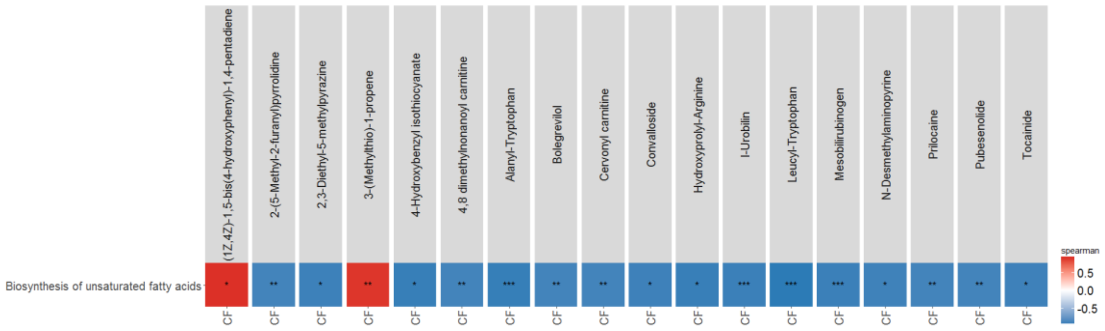

(iv) Alpha-linolenic acid metabolism

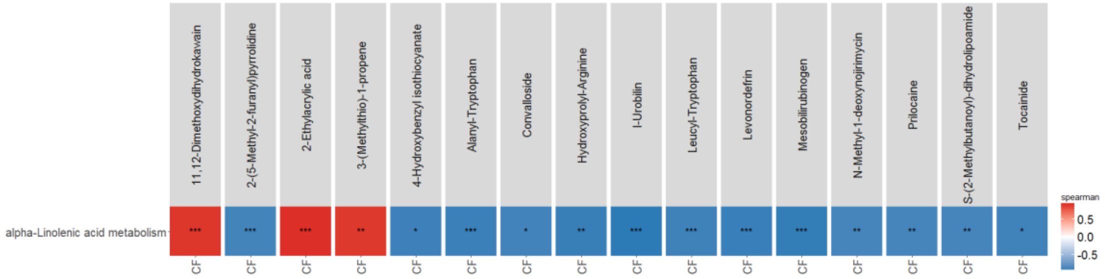


(v) Tryptophan metabolism

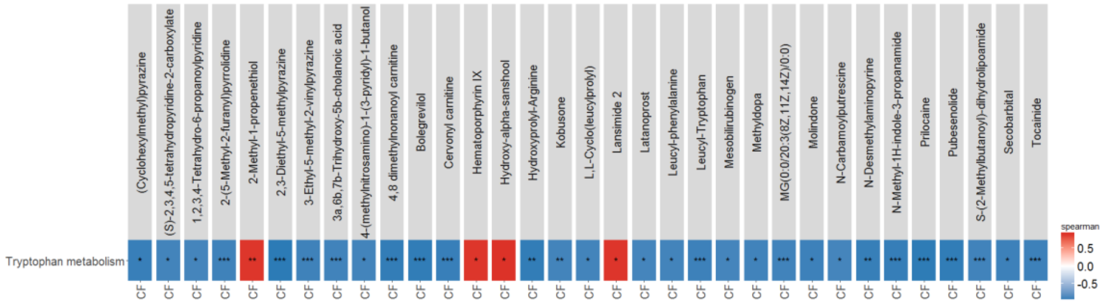


## B. Negative Ion Mode

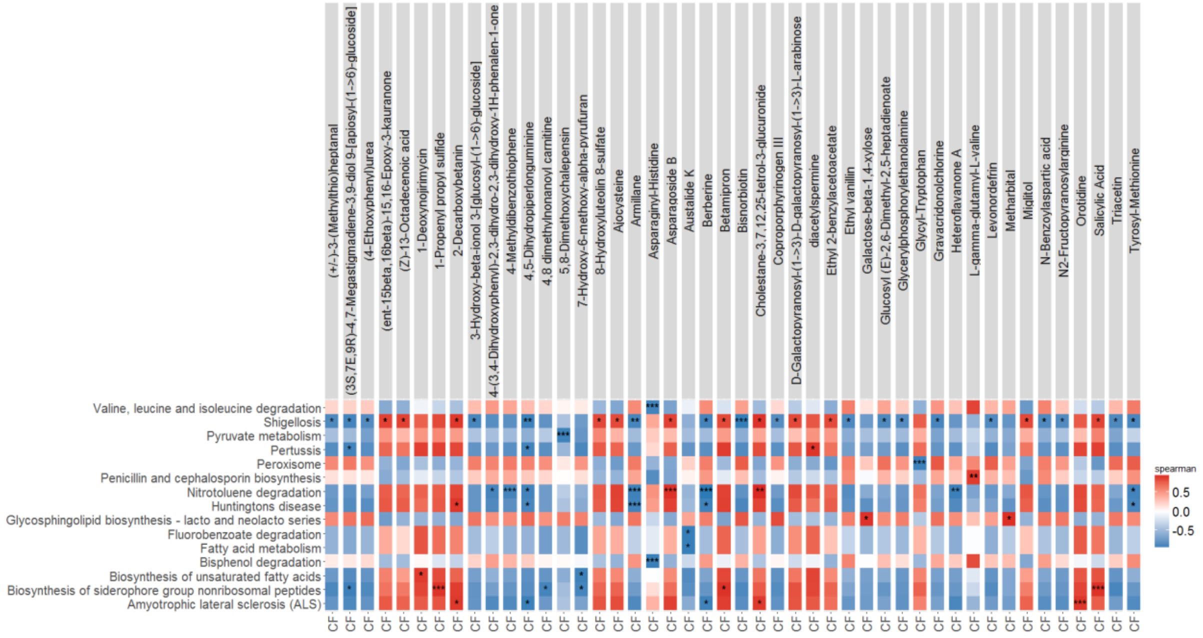


**Supplementary Figure 12**. Correlations between the normalised abundances of metabolites in positive ion mode (**A**) or negative ion mode (**B**), and the relative abundances of KEGG pathways (significant pathways identified in Manuscript Table 3) in children with CF. Spearman correlations corrected for multiple testing (B-H correction). 801 significant correlations ($q<0.05$) were identified in the positive ion mode analysis (Supp. Data. 6), therefore section **A** presents only pathways related to antioxidants, fatty acids and amino acids. *, $q<0.05$; **, $q<0.01$; ***, $q<0.001$.

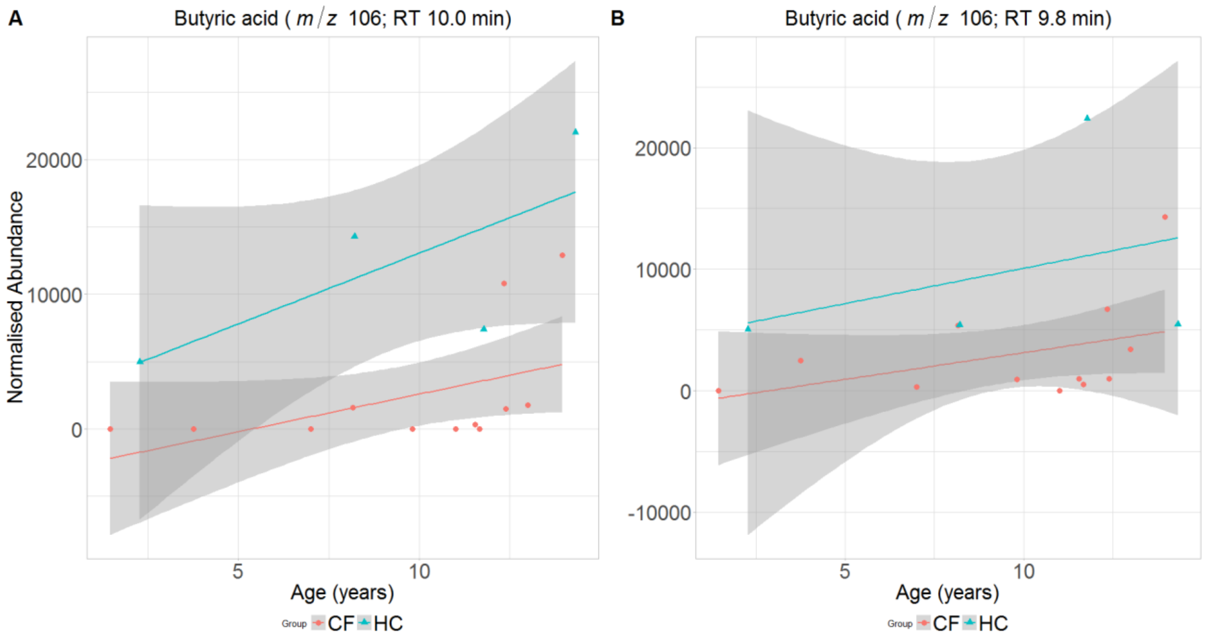


**Supplementary Figure 13**. Scatterplots of the normalised abundance of butyric acid metabolites against age in CF (n=12) and HC (n=4) samples. Cohort mean and 95% confidence intervals are constructed from generalised linear models and presented as lines and shaded regions, respectively. Metabolite *m/z* 106, RT 10.0 min (A) significantly lower in CF compared to HC (normalised abundance estimate (SE) -10,134.4 (2,631.8), p=0.002). Metabolite *m/z* 106, RT 9,8 min (B) significantly lower in CF compared to HC (normalised abundance estimate (SE) -6,841.5 (3,011.4), p=0.04).

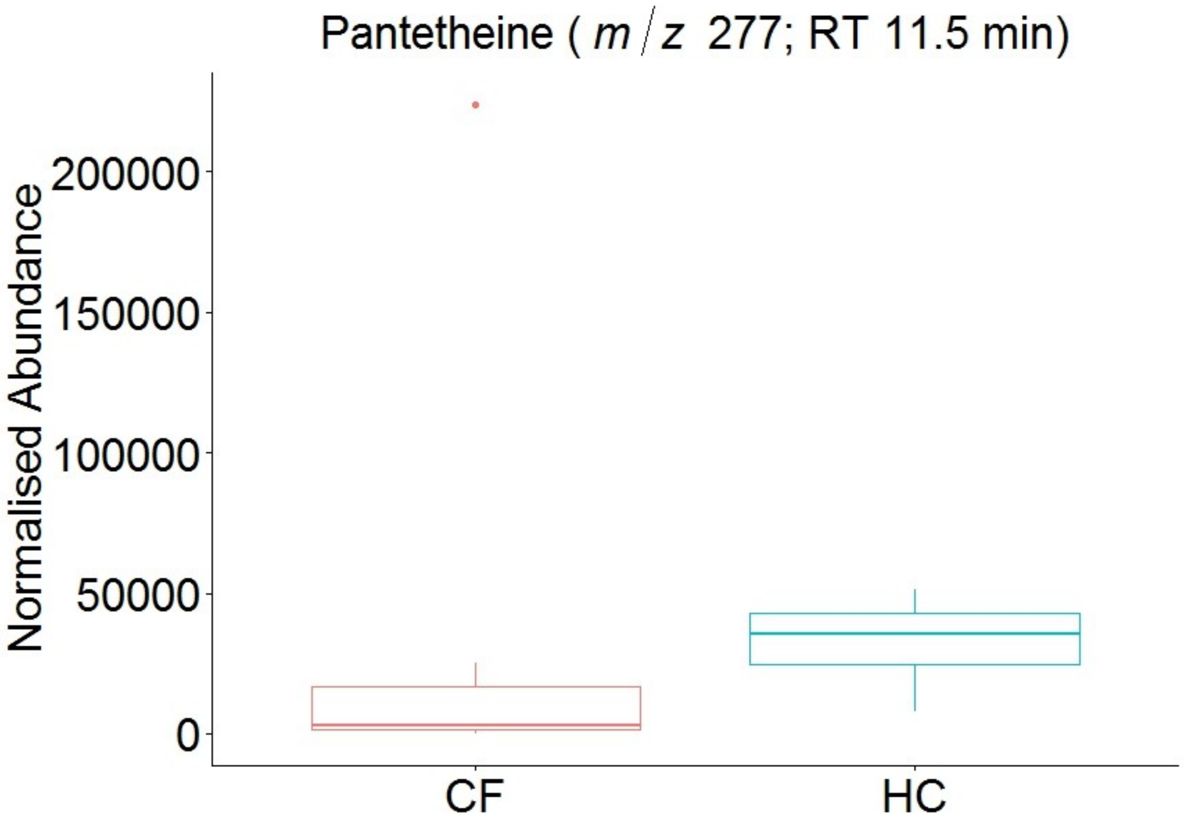


**Supplementary Figure 14**. Boxplot of normalised abundance of pantetheine in CF (n=12) and HC (n=4) samples. Metabolite *m/z* 277, RT 11.5 min significantly lower in CF compared to HC (normalised abundance median (IQR) 2,795 (1,343-16,628) vs. 35,418 (24,798-43,056), p=0.045).

**Supplementary Dataset 1**. Meta data

Coffey_Tax4Fun_Supplementary_Data_metadata.csv

**Supplementary Dataset 2**. zOTU table

Coffey_Tax4Fun_Supplementary_Data_otus.csv

**Supplementary Dataset 3**. KEGG pathways table

Coffey_Tax4Fun_Supplementary_Data_pathways.csv

**Supplementary Dataset 4**. Normalised abundance of metabolites (positive ion mode) identified using the Human Metabolome Database (HMDB).

Coffey_Tax4Fun_Supplementary_Data_known_metabolites_Pos_mode.csv

**Supplementary Dataset 5**. Normalised abundance of metabolites (negative ion mode) identified using the Human Metabolome Database (HMDB).

Coffey_Tax4Fun_Supplementary_Data_known_metabolites_Neg_mode.csv

**Supplementary Dataset 6**. Significant spearman correlations ($q<0.05$) between: (i) bacterial genera (significant genera identified in Table 2) and metabolites (positive ion mode) (1st tab); (ii) bacterial genera (significant genera identified in Table 2) and metabolites (negative ion mode) (2nd tab); (iii) KEGG pathways (significant pathways identified in Table 3) and metabolites (positive ion mode) (3rd tab); and (iv) KEGG pathways (significant pathways identified in Table 3) and metabolites (negative ion mode) (4th tab).

Coffey_Tax4Fun_Supplementary_Data_Correlations_16S-KEGG_Metabolites.xlsx

**Supplementary Dataset 7**. Metabolites related to propanoate and/or butanoate metabolism which were searched for in untargeted metabolomics data.

Coffey_Tax4Fun_Supplementary_Data_SCFA_pathways_searched.csv

**Supplementary Dataset 8**. Normalised abundance of metabolites (positive ion mode) related to propanoate and/or butanoate metabolism.

Coffey_Tax4Fun_Supplementary_Data_SCFA_Pos_mode.csv

**Supplementary Dataset 9**. Normalised abundance of metabolites (negative ion mode) related to propanoate and/or butanoate metabolism.

Coffey_Tax4Fun_Supplementary_Data_SCFA_Neg_mode.csv
